# Diagnostic Implications of Multi-Cancer Early Detection Testing in the Investigation of Cancer Symptoms: An Exploratory Retrospective Analysis of the SYMPLIFY Study

**DOI:** 10.1016/j.lanepe.2026.101720

**Published:** 2026-05-28

**Authors:** Ashley Jackson, Pradeep S. Virdee, Sharon Tonner, Jason L. Oke, Rafael Perera, Kaveh Riahi, Sara Hiom, Harpal Kumar, F.D. Richard Hobbs, Mark R. Middleton, Brian D. Nicholson

**Affiliations:** a Nuffield Department of Primary Care Health Sciences, University of Oxford, Oxford, UK; b Department of Medicine, University of Ottawa, Ottawa, Ontario, Canada; c GRAIL Bio UK, Ltd., London, UK; d Department of Oncology, University of Oxford, Oxford, UK

**Keywords:** MCED, Early diagnosis, Cancer, False positive

## Abstract

**Background:**

The observational SYMPLIFY study reported the accuracy of a multi-cancer early detection (MCED) test in a referred symptomatic population. We explore how the MCED test may contribute to a faster or more efficient diagnosis if acted upon.

**Methods:**

We reviewed all cancers diagnosed in SYMPLIFY (ISRCTN10226380) using data collected at study sites plus two years of cancer registry data. All cancer diagnoses were classified based on the congruence between the participant's symptoms, diagnostic referral pathway, MCED test cancer signal origin (CSO) prediction, cancer site, and time to diagnosis.

**Findings:**

There were 533 cancers diagnosed among 5461 (9.8%) evaluable participants in SYMPLIFY during the 2-year follow-up period. Among the 79 participants with an apparent false positive test result in the original SYMPLIFY study, 28 (35%) were diagnosed with cancer based on cancer registry data, increasing the MCED PPV to 84.2% (80.1–87.6). In aggregate, only one of the 28 additional patients had a cancer diagnosed that was incongruent with a predicted MCED CSO. Among the 5014 patients with an apparent true negative MCED test result, 113 (2%) received a subsequent cancer diagnosis. In 101 (19%) of the 533 cancers diagnosed, the MCED test result might have contributed to a more efficient diagnosis had it been used to inform the clinical work-up. Conversely, 49 (9%) cancers might have taken longer to diagnose if the MCED test result alone had been used in the diagnostic process, directing investigations based upon an incorrect CSO prediction.

**Interpretation:**

These exploratory findings demonstrate a substantially higher rate of cancer diagnoses in symptomatic participants originally classified with a false positive MCED test result than those originally classified as true negative. We show how MCED tests have the potential to assist clinical decision making, which may in turn lead to a timelier cancer diagnosis for one fifth of cancers diagnosed.

**Funding:**

GRAIL Bio UK, Ltd. NIHR.

**Research in context**

**Evidence before this study**

We searched PubMed from database inception to Sep 4, 2025, for “multi-cancer screening” [title and abstract] OR “multi-cancer detection” [title and abstract] AND “symptoms” [all fields] (and related terms) without language restrictions. In addition to evidence that was already identified in the original SYMPLIFY study, we identified the PATHFINDER study, a prospective cohort study that assessed a blood-based multi-cancer early detection (MCED) test for cancer screening in asymptomatic adult patients. The study returned MCED test results to participants’ doctors to aid diagnostic evaluation. The study found that less than 1% of study participants had a false positive test result, with 38% of patients who had a cancer signal detected eventually diagnosed with cancer. Additionally, the study found that the first cancer signal origin (CSO) was correct in 85% of patients, and the first or second was correct in 97% of patients. PATHFINDER demonstrated the feasibility of blood based MCED tests for cancer screening and the accuracy of MCED CSO for guiding clinical investigations. The SYMPLIFY study also demonstrated the feasibility of using a blood based MCED test but in a symptomatic population, with a sensitivity of 66.3% (95% CI 61.2–71.1) and specificity of 98.4% (98.1–98.8). Like PATHFINDER, the SYMPLIFY study demonstrated high accuracy of the MCED CSO at 85.2%. We also identified updated findings of the DETECT-A study which followed-up patients deemed false positive in the original study for a median of 3.6 years and found that the annual incidence of cancer in the false positive group was not elevated, at 1.0% (95% CI 0.2%–2.8%).

**Added value of this study**

Uncertainties remain about where MCED tests may be integrated within diagnostic pathways for symptomatic patients and the impact of a false positive MCED test result in this population. Through extended 2-year follow-up in cancer registry data, we demonstrate a 16-fold higher incidence of cancer in symptomatic patients with a false positive test result on site collected data in the 9 months following study enrolment than those classified as true negative. To our knowledge, this is the first study to investigate the residual cancer risk in symptomatic patients who were considered to have had a false positive MCED test result based on standard of care clinical evaluation. We also highlight three distinct ways that acting on an MCED test result during the diagnostic work-up of symptoms concerning for cancer could lead to more efficient cancer diagnoses, including improving the time to diagnosis, the investigations used, or choice of referral pathway. We outline instances where the utilisation of an MCED test result alone could lead to delays in diagnosis if other clinical factors, such as symptoms were ignored. We identify cancer sites and symptoms that may particularly benefit from the use of an MCED test in the work-up of suspected cancer, such as in differentiating between upper and lower GI cancers.

**Implications of all the available evidence**

Our results highlight an increased incidence of cancer in the year following study enrolment in participants with an initially false positive test result that may justify broader initial cancer investigations to enable earlier cancer diagnosis. Further work is needed to understand whether this elevated cancer incidence extends beyond the 2-year period to help determine for how long and how often patients with a false-positive MCED test result should be monitored for undiagnosed cancer and whether retests are necessary. Further, our results outline how MCED tests may aid clinicians with decisions regarding the diagnostic work-up of symptoms of suspected cancer to lead to more efficient diagnoses.

## Introduction

Globally, cancer presents a significant health burden, with estimates suggesting that approximately half of all people will receive a cancer diagnosis during their lifetime.^1^ When cancer is detected early there are a greater number of, and more effective, treatment options available, resulting in better survival.^2^ However, currently approximately half of all cancers are diagnosed at an advanced stage, resulting in high morbidity and mortality.^3^ Many countries are making early cancer detection a priority. A key element of the Faster Diagnosis Standard (FDS) of the English National Health Service is for 75% of patients to receive a cancer diagnosis within 28 days of a cancer referral.^4^^,^^5^ The Faster Diagnosis Framework also highlights rapid investigation of patients with non-specific symptoms (NSS) that are associated with many cancers.^4^^,^^6^ Patients are now referred to NSS pathways and undergo a broad set investigations, or are referred on to site-specific cancer pathways if appropriate.^7^ Although these pathways have improved the time to diagnosis, concerns remain about the residual risk of cancer in patients when no cancer is diagnosed after urgent referral.^8^^,^^9^

Multi-cancer early detection (MCED) tests are being investigated for their potential in improving cancer diagnosis by screening asymptomatic people or triaging symptomatic patients. MCEDs measure biological markers, such as circulating tumour DNA (ctDNA) and proteins, shed by cancer cells that are present in the blood.^10^^,^^11^ Currently, most studies have looked at the effectiveness of MCED tests for screening.^12^, ^13^, ^14^, ^15^, ^16^ The recent observational SYMPLIFY study demonstrated the potential for using an MCED test (Galleri®, GRAIL) in symptomatic people referred for cancer investigation, with an overall sensitivity of 66.3% (95% CI 61.2–71.1), specificity 98.4% (98.1–98.8), positive predictive value (PPV) 75.5% (95% CI 70.5–80.1), and negative predictive value (NPV) of 97.6% (97.1–98.0). SYMPLIFY illustrated how an MCED could inform clinical decision making by guiding the choice of definitive cancer testing, especially when initial guideline directed investigations did not identify cancer. Further investigation was needed to understand how MCED tests may aid clinicians in investigating symptoms that could be caused by cancer to reach a timelier cancer diagnosis.

In this study we reviewed all cancers reported in the national cancer registry within two years of enrolment in SYMPLIFY. We compared the rate of subsequent cancer diagnoses in participants who had a false positive MCED test result and those with a true negative MCED test result after symptom directed cancer investigation. We also analysed MCED test results, presenting symptoms, referral pathways selected, and the time to diagnosis in all participants diagnosed with cancer. Our aim was to identify whether MCED testing could lead to diagnostic efficiencies by reducing diagnostic delays, avoiding unnecessary investigations, and aiding clinicians to choose the most appropriate cancer investigations, while also investigating potential diagnostic delays associated with MCED test results.

## Methods

### Study design and participants

This work builds on the previous prospective observational SYMPLIFY cohort study (ISRCTN10226380) which evaluated an MCED test in symptomatic patients in England and Wales. The study was able to evaluate 5461 participants referred with symptoms concerning for cancer to 44 hospitals in England and Wales by their primary care clinician for rapid cancer investigation to a NSS pathway, or to a standardised gynaecological, lung, upper, or lower gastrointestinal urgent cancer two-week-wait (2WW) pathway.^17^ Recruiting hospital sites were asked to review the local hospital records for all participants at 3 months post-enrolment and enter data into electronic case report forms (CRFs), with those unresolved at the 3-month mark undergoing a second review at 9 months.^17^

### Procedures

Included cancers were identified from data collected locally at hospital sites and recorded in the SYMPLIFY CRF, and from the national cancer registries in England and Wales. The registry data were assessed for any cancers among SYMPLIFY participants that were registered but not reported in the SYMPLIFY CRF. For cancers that were reported in both the CRF and cancer registry, the relevant CRF data were used.

Only cancers with an ICD-10 codes starting with ‘C’ or D45-D46.9, D47.3, D47.4 were included. ICD-10 codes of C44 and C519 were excluded as non-melanoma skin cancers were not included in the original SYMPLIFY study.^17^ Data regarding the symptoms present at the time of urgent referral for investigation of cancer were also recorded for each patient in the SYMPLIFY CRF and were obtained for this study. MCED test results from the original SYMPLIFY study were obtained for each participant who had been diagnosed with cancer. Test results included a cancer signal detection (CSD, yes/no) and the top two predicted cancer signal origins (CSO).^17^

All cancers identified in either the CRF or the respective national cancer registry were reviewed for their congruency between the cancer diagnosis and the initial presenting symptoms, the referral pathway, and the MCED CSO results. The ICD-10 code was used to identify the location of the malignancy. Each case was reviewed by two clinicians.

A cancer was considered congruent with the symptoms at presentation if the symptoms that were present could be reasonably explained by the diagnosis, which was informed by the National Institute for Health and Care Excellence (NICE) Guideline 12 suspected cancer recognition and referral (NG12). In cases where participants had a positive MCED test, the MCED 1st CSO and diagnosed cancer site had to be the same to be considered congruent. If the MCED test reported no cancer signal detected, then the MCED was considered incongruent with the diagnosis. A diagnosis was considered congruent with the referral pathway if the cancer site was one which could be reasonably identified by the investigations usually conducted by that referral pathway. All cancer sites were congruent with referral to an NSS pathway, given the wide breadth of investigations and cancers that can be diagnosed through these pathways. We had multiple independent clinical reviewers classify each congruency case until an agreement was made, utilising both the NG12 guidelines and cancer literature, allowing for internal consistency. The time to diagnosis was calculated for each cancer by subtracting the date of diagnosis from the date of enrolment of the participant in the study.

The MCED test result was considered to have the potential to enhance diagnostic efficiency when the final diagnosis was congruent with the MCED predicted CSO. However, in practice, some cancers in this group would not derive a meaningful benefit in diagnostic efficiency if the MCED had been used, because the diagnosis was clear from clinical presentation and was carried out in a timely manner. Thus, we further identified three distinct and potentially meaningful benefits the MCED test may have on the diagnostic process. First, any cancer in which the diagnosis was congruent with the MCED CSO but not the referral pathway. Second, the use of the MCED may have simplified and sequenced investigations when the MCED test result was congruent with the final diagnosis in patients referred to an NSS pathway. Third, any cancer in which the time to diagnosis was longer than the 28-day benchmark as the MCED could have directed appropriate cancer investigations sooner.

We identified scenarios where diagnostic delays could have resulted from MCED test results when the CSO was incorrect, potentially resulting in incorrect cancer investigations if the MCED CSO alone were used whilst ignoring other clinical factors. In the previous SYMPLIFY study, the authors concluded that, other than in patients with suspected upper GI cancers, a negative MCED test was not sufficient to reduce the risk of cancer sufficiently to rule out further cancer investigation in a referred symptomatic patient.^17^ Thus, for this analysis, we only considered the MCED result as a source of potential diagnostic delay when the MCED test was positive but the CSO was not congruent with the final diagnosis.

### Outcomes

The primary study objective was the rate of subsequent cancers in the 24-months post-study enrolment in the ostensibly False Positive and True Negative groups from the original SYMPLIFY study. Secondary objectives included a descriptive analysis of the cancers diagnosed in the 24 months post-study enrolment based on their congruency between the presenting symptoms, referral pathway, and MCED test results to determine which cancers may have benefitted from the MCED test being used in the diagnostic work-up for suspected cancer.

The main objective stated in the statistical analysis plan for this pre-planned analysis was “to assess the diagnostic accuracy of the MCED test using diagnoses within 2 years.” The plan also stated that “we may describe whether the additional registry cancers are congruent with presenting symptoms, referral pathway chosen, and the initial MCED result (CSD and CSO),” and “we may summarise the time from registration to diagnosis, site, and stage of cancers.” Together these analyses permitted our assessment of diagnostic efficiency. However, we did not explicitly describe these additional analyses as the evaluation of diagnostic efficiency in the statistical analysis plan.

### Data analysis

The time to diagnosis was displayed as the median and IQR of all cancers and among groups based on cancer site, stage, MCED test result, and congruency. Congruency and potential improvements in diagnostic efficiency attributable to the MCED test were displayed as the number and percentage of total cancers that were classified in each group. Further analyses were conducted on cancers that were congruent with the presenting symptoms but not the referral pathway to characterise the cancer sites and referral pathways common among this group. Cancer diagnoses that may have benefited from the MCED test result were reported for each distinct benefit, and as a percentage of the total cancers with the given characteristic.

The congruency of the MCED test result was also reported for cancer cases classified as false positive and true negative in the SYMPLIFY study. If the cancer diagnosis was congruent with at least one of the symptoms, pathway, or MCED CSO then the cancer was classified to have likely been related to the initial presentation. Those that were not congruent with any of the symptoms, pathway, or MCED CSO were considered unlikely to have been related to the initial clinical presentation (i.e., potentially an incidental finding).

Most analyses throughout this study were conducted on a cancer level (e.g., if a patient had two cancer diagnoses, these were considered separately) to account for congruency between symptoms, pathway, MCED CSO, and diagnosis. The exception was the analysis of false positive and true negative test results, as these analyses were conducted on a patient level to determine the cancer conversion rate in each group of participants. All analyses were completed in Stata version 17.0.

### Ethics approval

All patients provided written informed consent, and the study was undertaken in accordance with the Declaration of Helsinki. The protocol was approved by the National Research Ethics Service (21/LO/0456–London Central) and complied with UK regulations as outlined in the original SYMPLIFY study.^1^ Consent was also gained from participants for the SYMPLIFY team to retrieve data held in central national databases, such as the cancer registry, for 2 years following their enrolment into the study.

### Role of the funding source

The lead investigators (AJ, BDN, MRM), GRAIL, NHS England, and the University of Oxford designed the current study together. The University of Oxford sponsored SYMPLIFY and was responsible for data collection, data analysis, and data interpretation. GRAIL provided the results of the MCED test, but had no role in the analysis and interpretation of the data. GRAIL authors participated in the review of the statistical analysis plan for SYMPLIFY and of the analyses that flowed from it. GRAIL authors contributed to the writing of the report.

## Results

The median age at registration of the 5461 evaluable participants in SYMPLIFY was 61.9 years (IQR 53.4–73.0), 66.1% (3609) of the participants were female, 4370 (80.0%) were recruited in England and 1091 (20.0%) were recruited in Wales.^17^ There were 533 cancers diagnosed in 509 participants throughout the follow-up period. There were 380 cancers diagnosed among 368 patients in the SYMPLIFY CRF, which were reported in the original SYMPLIFY study. The remaining 153 cancers diagnosed among 141 participants were not reported but identified through the national cancer registries. The median number of days between enrolment and diagnosis was 33 (IQR = 10–98). Table 1 highlights the characteristics of all 533 cancers included in the study.

**Table 1:** Characteristics of all cancers.

Characteristic	Number (%)	Median days to diagnosis (IQR)
Whole cohort	533	33 (10–98)
Patient demographics		
Age group		
50–59	87 (16)	38 (9–132)
60–69	125 (23)	27 (10–86)
70–79	198 (37)	35 (11–108)
80–89	85 (16)	22 (9–91)
90+	12 (2)	63.5 (14.5–239)
<50	26 (5)	20 (6–71)
Sex		
Female	281 (53)	37 (12–110)
Male	252 (47)	29 (8–93)
Ethnicity		
Non-white	15 (3)	97 (42–540)
White	518 (97)	32 (10–95)
Cancer characteristics		
Cancer stage		
Stage I	163 (31)	59 (14–254)
Stage II	85 (16)	51 (15–106)
Stage III	138 (26)	21 (7–56)
Stage IV	106 (20)	18.5 (7–63)
Unknown	41 (8)	63 (27–228)
Cancer site		
Anus	5 (1)	29 (7–32)
Bladder and urothelial tract	22 (4)	68.5 (10–324)
Bone and soft tissue	1 (0.2)	42 (−)
Breast, female	28 (5)	427 (121–553.5)
CNS	2 (0.4)	225.5 (46–405)
Cancer of unknown primary	3 (0.6)	41 (22–139)
Cervix	5 (1)	10 (8–51)
Colorectal	159 (30)	16 (4–57)
Gallbladder	1 (0.2)	16 (−)
Head and neck	2 (0.4)	34.5 (34–35)
Liver, bile duct	9 (2)	47 (21–66)
Lung, trachea, and bronchus	106 (20)	15 (6–69)
Lymphoid	25 (5)	68 (24–163)
Melanoma of skin	8 (2)	218 (32–415.5)
Myeloid	1 (0.2)	391 (−)
Oesophagus	25 (5)	13 (5–52)
Other	17 (3)	45 (27–81)
Ovarian	20 (4)	43.5 (19–125)
Pancreas	19 (4)	44 (15–99)
Plasma cell	5 (1)	210 (195–585)
Prostate	28 (5)	166 (88–516)
Stomach	5 (1)	9 (0–27)
Thyroid	3 (0.6)	208 (63–495)
Uterus	34 (6)	45.5 (16–73)
Referral pathway		
Gynae 2WW	81 (15)	68 (17–157)
Lower GI clinic	213 (40)	29 (10–103)
Lung 2WW	104 (20)	14 (6–34)
Rapid diagnostic centre	49 (9)	51 (19–120)
Upper GI 2WW	86 (16)	53.5 (19–299)
Time to diagnosis		
Within 28 days of enrolment	252 (47)	9 (1–16)
>28 days after enrolment	281 (53)	93 (56–362)
Present in CRF/registry		
CRF only	45 (8)	21 (7–69)
Registry only	154 (29)	323.5 (90–548)
Both	334 (63)	20 (7–50)

Presented as the number and percentage of total cancers that exhibit each quality. Time between diagnosis and enrolment is displayed as the median with the IQR. Cancer sites labelled “Other” were used in instances where the cancer site was incomplete or ambiguous, such as instances where the cancer was listed as “Neoplasm, Not Otherwise Specified.”

There were 79 participants in the original SYMPLIFY study that had a positive MCED test result but did not have a cancer reported in the SYMPLIFY CRF (false positive). Among these, 28 (35%) had a cancer identified in the respective national registry within 2 years of study enrolment, accounting for 31 cancers in total, and increasing the MCED PPV to 84.2% (80.1–87.6). There were 5014 patients who had a negative MCED test (i.e., no cancer signal detected) who did not have a cancer reported in the SYMPLIFY CRF (true negative), of which 113 (2%) had a cancer reported in the national cancer registry within 2 years of study enrolment. This accounted for 115 total cancers.

**Fig. 1:**
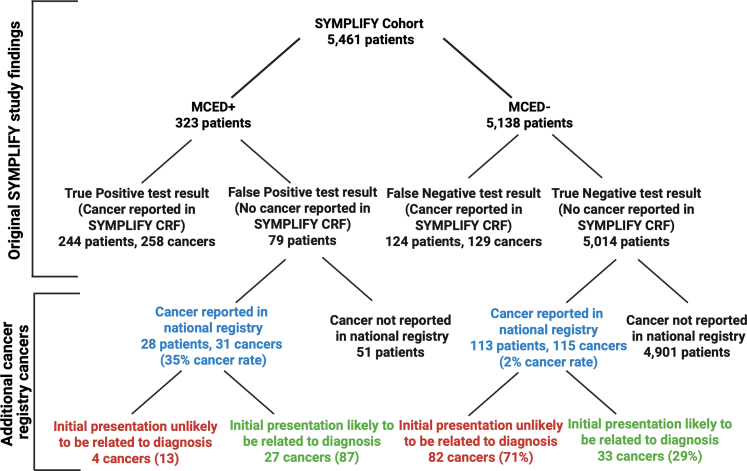
**Breakdown of patients and cancers in the original and following the addition of subsequent registry cancers**.

We found that 27 (87%) of the 31 cancers diagnosed in what was previously the false positive group were likely to be related to the initial presentation, compared to only 33 (29%) of the 115 cancers in the previous true negative group (Fig. 1). Among the 31 cancers in the false positive group, 24 (77%) were congruent with the first MCED CSO. An additional three were congruent with the second CSO. Furthermore, two of the four cancers that was not congruent with the MCED CSO occurred in patients with two cancers diagnosed, where one of the cancers was congruent with the MCED test. Therefore, in aggregate, only one of the 28 additional patients had a cancer that was incongruent with one of the reported CSOs. Of the 28 participants initially classified with a FP MCED that were subsequently reported to have a cancer diagnosed in the cancer registry, 15 (54%) were female (Supplementary Table S1). 16/28 (57%) were diagnosed with cancer within 9 months of enrolment. 8 of the 16 were diagnosed with cancers that were incongruent with the diagnostic pathway chosen based on their presenting symptoms, but the MCED Cancer Signal Origin (CSO) call was correct. The other 8 were referred to the correct diagnostic pathway for the cancer diagnosed, suggesting the cancer was missed by standard of care investigations or data entry errors during SYMPLIFY. 12/28 (39%) were diagnosed with cancer 10–24 months following enrolment (Supplementary Table S2). 7 of the 12 were diagnosed with pathway incongruent cancers but the MCED CSO was correct. For the remaining 5, the pathway initially chosen was appropriate for the cancer registered, suggesting the cancer was undetectable on initial standard of care investigation.

289 (54%) of the 533 cancers occurred in patients who received a positive MCED test result. Overall, 240 (45%) cancers were congruent with the MCED CSO, 389 (73%) with symptoms, and 358 (67%) with the referral pathway. The time to diagnosis was shortest for those cancers that were congruent with the symptoms, referral pathway, and MCED CSO (median = 14, IQR = 4–31) (Table 1).

There were 101 (19%) cancers that might have had a meaningful improvement in diagnostic efficiency had the MCED test result been used in the diagnostic process. Among these, 39 (39%) were congruent with the MCED CSO but the referral pathway was incongruent with the final diagnosis (Table 2). There were 18 (18%) cancers that were congruent with the MCED test result, and the patient was referred to an NSS pathway. The remaining 44 (44%) were congruent with the symptoms, pathway, and MCED CSO but did not achieve the outlined standard of diagnosis within 28 days of urgent referral and did not meet the criteria for the other two aforementioned groups. There were 49 (9%) cancers that may have experienced diagnostic delays if the MCED test result alone had been used in the diagnostic process due to an incorrect CSO guiding the diagnostic decisions and ignoring other clinical factors (Table 2).

**Table 2:** Characteristics of cancers diagnosed based on MCED results and congruency with symptoms, pathway, and MCED CSO.

Characteristics	Number (%)	Median Days to Diagnosis (IQR)
MCED results		
MCED Result		
Negative	244 (46)	81.5 (25–342.5)
Positive	289 (54)	17 (6–45)
SYMPLIFY Classification		
False negative	129 (24)	37 (14–73)
False positive	31 (6)	120 (35–460)
True negative	115 (22)	405 (147–583)
True positive	258 (48)	15 (5–35)
Congruency with cancer diagnosed		
Symptoms		
No	144 (27)	217.5 (66–538.5)
Yes	389 (73)	20 (7–55)
Pathway		
No	175 (33)	132 (47–495)
Yes	358 (67)	18 (6–51)
MCED 1st CSO		
No	293 (55)	68 (19–272)
Yes	240 (45)	16 (6–41.5)
MCED 1st or 2nd CSO		
No	276 (52)	71.5 (22–289.5)
Yes	257 (48)	16 (6–44)
Overall Congruency		
Symptoms, Pathway, and 1st MCED CSO	201 (38)	14 (4–31)
None	115 (22)	273 (89–546)
Symptoms and Pathway only	143 (27)	24 (8–68)
Symptoms and 1st MCED CSO only	24 (5)	52 (27.5–90)
Pathway and 1st MCED CSO only	–	–
Symptoms only	21 (4)	68 (34–103)
Pathway only	14 (3)	112 (56–323)
1st MCED CSO only	15 (3)	44 (19–546)

Presented as the number and percentage of total cancers. Days to diagnosis is demonstrated as the median with the IQR.

There were 252 (47%) cancers diagnosed throughout the study that achieved the benchmark of diagnosis within 28 days of urgent referral for suspected cancer (Table 1). Among the cancers that may have had improved diagnostic efficiency due to the use of the MCED test, 79 (78%) were diagnosed outside of the 28-day standard (Table 3). If the use of the MCED test allowed all these cancers to be diagnosed within the 28-day standard, then the rate of cancers diagnosed within the target time frame in this study could have improved from 252 (55%) to 331 (62%).

**Table 3:** Potential meaningful improvements in diagnostic efficiency associated with MCED use and potential for impact on diagnoses taking over 28 days.

MCED impact	Number of cancer diagnoses (% of total)	Number of cancer diagnoses taking >28 days n (%)
Total potential improvements in diagnostic efficiency		
MCED correctly directs cancer investigation (correct CSO)	240 (45)	79/240 (33)
Potential meaningful improvements in diagnostic efficiency		
MCED 1st CSO correctly directs referral when an incorrect referral pathway was chosen based on symptoms.	39 (7)	28/39 (72)
MCED 1st CSO correctly specifies the cancer site in patients referred for broad investigation of non-specific symptoms.	18 (3)	7/18 (39)
MCED 1st CSO could shorten the time to diagnosis for cancers with a time from referral to diagnosis >28 days.	44 (8)	44/44 (100)
Total meaningful improvements	101 (19)	79/101 (78)
Potential diagnostic delays		
Incorrectly directs investigations		
Incorrect 1st CSO	49 (9)	22/49 (45)
Incorrect 1st and 2nd CSO	32 (6)	

When looking at specific cancer sites, there were 23 types of cancers diagnosed throughout the SYMPLIFY study, of which 16 (70%) may have had improved diagnostic efficiency had the MCED test result been used in the diagnostic work-up (Table 4). As for stage, 58% (59) of all cancers that could have had a meaningful benefit in improving diagnostic efficiency were stage I-III cancers (Table 4).

**Table 4:** Characteristics of cancers with potential meaningful benefit of MCED.

Characteristic	Overall cancers (N)	Meaningful benefit cancers n (%)
Pathway		
Gynae	81	18 (18)
Lower GI	214	32 (32)
Lung	106	13 (13)
NSS	49	18 (18)
Upper GI	86	20 (20)
Cancer site		
Anus	5	–
Bladder and urothelial tract	22	3 (3)
Bone and soft tissue	1	–
Breast, female	28	4 (4)
CNS	2	–
Cancer of unknown primary	3	2 (2)
Cervix	5	–
Colorectal	160	34 (34)
Gallbladder	1	1 (1)
Head and neck	2	1 (1)
Liver, bile duct	9	3 (3)
Lung, trachea, and bronchus	108	10 (10)
Lymphoid	25	8 (8)
Melanoma of skin	8	–
Myeloid	1	–
Oesophagus	25	2 (2)
Other	17	2 (2)
Ovarian	20	8 (8)
Pancreas	19	11 (11)
Plasma cell	5	2 (2)
Prostate	28	1 (1)
Stomach	5	–
Thyroid	3	–
Uterus	34	9 (9)
Cancer stage		
Stage I	164	13 (13)
Stage II	85	21 (21)
Stage II	140	25 (25)
Stage IV	106	37 (37)
Unknown	41	5 (5)

Cancers with a meaningful benefit of the MCED include one of the three scenarios: First, any cancer in which the diagnosis was congruent with the MCED CSO but not the referral pathway. Second, the use of the MCED may have simplified and sequenced investigations when the MCED test result was congruent with the final diagnosis in patients referred to an NSS pathway. Third, any cancer in which the time to diagnosis was longer than the 28-day benchmark as the MCED could have directed appropriate cancer investigations sooner. Data are presented as the number and percentage of cancers with the specific characteristic among all the cancers with a potential meaningful benefit of the MCED (n = 101).

**Fig. 2:**
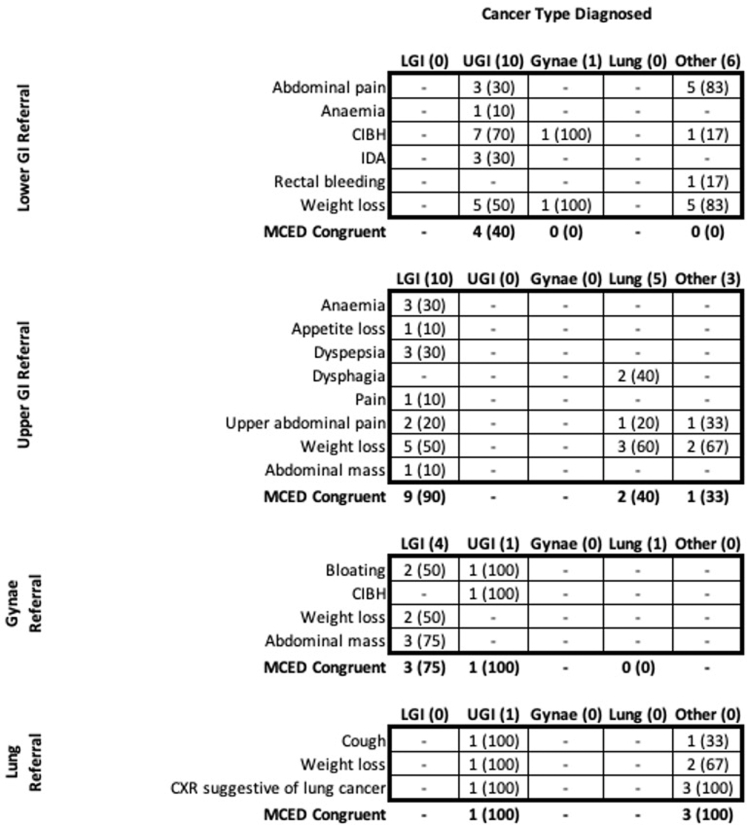
**Symptom profiles of cases for which symptoms were congruent with cancer diagnosis but patients were referred to a pathway incongruent with the cancer (n** = **45).** Values are presented as the number of patients with a given symptom and the percentage of total patients with the symptom. The final row for each referral pathway indicates the number and percentage of cancers within the group that were congruent with the MCED CSO. CIBH = change in bowel habit. IDA = iron-deficiency anaemia. CXR = chest x-ray. The list of symptoms is comprised of the 15 most common symptoms among patients in the study with the addition of abdominal mass and CXR suggestive of lung cancer.

There were 45 (8%) cancers where the symptoms the participant presented with were congruent with the final cancer diagnosis, but the pathway was not. Among these cancers 24 (53%) were congruent with the MCED CSO (Supplementary Table S1). The most common scenario was participants being referred to the lower GI pathway who were subsequently diagnosed with an upper GI cancer, and vice-versa (n = 20, 44%) (Fig. 2). Symptoms that were common amongst these incorrectly referred upper and lower GI cancers included changes in bowel habit and weight loss (Fig. 2).

## Discussion

We identified 28 (35%) participants who were originally classified as having a false positive MCED test result who had cancers identified in registry data within 2 years of study enrolment. This was a higher proportion than in the true negative group, where 113 (2%) participants had a cancer identified in registry data within 2 years. Although exploratory in nature, our findings suggest that among the 31 cancers diagnosed in 28 patients in the false positive group 27 (87%) could have been identified if cancer investigation had been directed by the MCED result.

We evaluated instances where the MCED may lead to more efficient cancer diagnoses. In doing so, we identified 101 (19%) cancers that might have had a more efficient diagnosis if the MCED test result had directed further cancer investigations in this symptomatic cohort. Using the MCED CSO may have improved the time to diagnosis, directed referral to a more appropriate urgent referral pathway, or informed the choice and sequencing of investigations for non-specific symptoms to reduce diagnostic delays, cost, and patient burden. With this, we identified that 16 of the 23 cancer types in the study demonstrated some benefit of the MCED test. The cancer types that did not demonstrate any benefit were largely rarer cancers with only a small prevalence. A larger study could better elucidate if these lower incidence cancers receive any diagnostic benefit from the MCED test.

We identified 49 (9%) cancers that may have experienced potential delays if the MCED test alone had been used in the diagnostic process due to the potential for referral to an incorrect pathway. We also identified 45 (8%) cancers that were referred to a pathway that was incongruent with the final diagnosis, despite having symptoms congruent with the identified cancer site. Among these, 25 (55.6%) were congruent with the MCED test result, indicating an opportunity for the MCED test to aid in more accurate referral. Although these represent intriguing findings of the potential role of MCED testing in aiding diagnostic efficiency, the exploratory nature of this research must not be understated. Still, the present work provides areas for further exploration regarding the potential impact of MCED testing on improving the efficiency of the cancer diagnostic pathway.

To our knowledge, this is the first study to follow-up symptomatic patients who received an MCED test beyond the conclusion of standard of care investigations. A strength of this study was the use of registry data, for which we have evidence that it is a comprehensive and reliable source for cancer diagnostic data.^18^ However, registry data are more limited in scope, due to standardised collection and reporting guidelines, which limited us in our ability to investigate issues of congruence between the registry and CRF datasets.

A limitation of our study is the potential ambiguity associated with determining whether a patient's symptoms are reasonably explained by their cancer diagnosis. Cancers can present in very different ways, and so it takes care to determine which symptoms are likely attributed to a cancer diagnosis and those that are not. We aimed to minimise this issue by having multiple independent clinical reviewers classify each case until an agreement was made, and by remaining consistent regarding which symptoms were aligned to each cancer, referring to national guidelines (NG12) and the cancer literature, which provided clear guidance regarding diagnostic standards.

The final limitation of our study is our assertion that participants who were referred to an NSS pathway and were congruent with the MCED CSO could have had a more efficient diagnosis based on the MCED test result. We suggest that patients who are referred to an NSS pathways could benefit from the use of the MCED test result by helping to triage investigations. However, there is limited data on the variation in the diagnostic approach taken by NSS pathways.^19^, ^20^, ^21^, ^22^ As such, our findings should therefore be taken as hypothesis-generating, with interventional research required to understand the clinical and economic impact of MCED testing on symptomatic patients.

The reduction in false positives from 79 in the original SYMPLIFY study to 51 in this analysis would result in the MCED PPV increasing from 75.5% (95% CI 70.5–80.1) to 84.2% (80.1–87.6) for cancer overall.^1^ A recent study conducted in the UK found that patients who undergo urgent investigation for cancer who do not have a cancer diagnosed at the time of referral have an increased probability of receiving a cancer diagnosis within 1–5 years post-referral, many of which were advanced-stage cancers.^8^ This risk was highest in the first 2 years after referral, and lower thereafter.^8^ The authors suggested that the subsequent cancer diagnoses may be due to increased overall cancer risk rather than missed diagnoses at initial referral.^8^ However, it is possible that at least some of these diagnoses, particularly advanced stage cancers in the 1–2 years following initial referral, may represent diagnoses that were missed or clinically undetectable at the time of referral. In the present study we have found that 87% of cancers diagnosed amongst the originally False Positive cohort within 2 years of study enrolment were related to the initial clinical presentation. This suggests that in clinical practice a positive MCED test serves as a biomarker for the early detection of existing cancer rather than being an indicator of there being an increased risk of developing cancer within 2 years. These findings together support further diagnostic work-up or potentially repeat MCED testing in patients considered to have a false positive MCED after initial cancer investigation, as previous evidence has shown that a persistent positive cancer signal on retest is associated with an increased risk of cancer diagnosis.^23^

Estimates suggest that around half of all suspected cancer patients present with non-specific symptoms that are not indicative of a particular cancer site.^6^ Previous studies have shown that non-specific symptoms, such as unexplained weight loss and fatigue, make triaging investigations and identifying referral pathways more challenging, leading to delays in diagnosis, and poorer cancer outcomes.^24^, ^25^, ^26^ In this study we found that 45 (8%) cancers were referred to a diagnostic pathway that was not congruent with the final diagnosis despite having symptoms that were indicative of their cancer. It is not only vague constitutional symptoms that represent referral challenges, but also more specific symptoms indicative of multiple potential cancer sites.^27^ Our findings illustrate that some patients who present with symptoms that could be indicative of multiple cancers may be referred to a particular pathway, receive a negative work-up, and then later be diagnosed with a similar cancer via a different referral pathway. In these patients, an MCED test result and corresponding CSO may have helped to direct patients to specific referral pathways or to identify which patients should be further investigated following an initial negative diagnostic work-up for cancer.

**Fig. 3:**
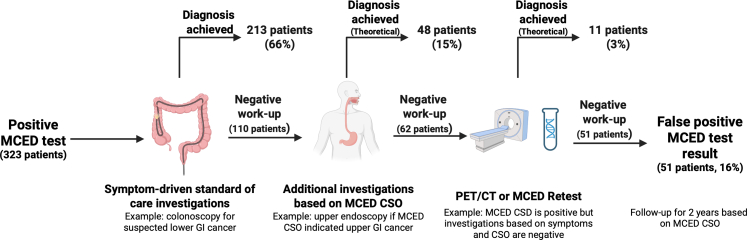
**Proposed pathway for investigating positive MCED test results in symptomatic patients with theoretical proportions based on study data.** All data in this figure are extrapolated from the current study data and are theoretical and illustrative.

Other MECD tests in development which do not have a CSO reporting capability propose using PET-CT to localise the cancer when a cancer signal is detected. However, PET-CT is not widely available in many health systems highlighting the need for robust health-system-specific health-economic analyses to inform the optimal investigative strategy to follow-up and rule-out cancer in MCED tested patients. We were able to assess whether the MCED might augment the standard of care provided for patients with symptoms of cancer in the NHS context in England and Wales. We suggest an alternative approach integrating the MCED into existing diagnostic pathways adding investigations to standard of care investigations if the CSO suggests a cancer site incongruent with the presenting symptoms and associated referral guideline criteria. A full-body work-up would be instigated only if investigations based on symptoms and CSO were negative, but the original CSD was positive (Fig. 3). Alternatively, a repeat MCED could be offered leading to further initial work-up based on the CSO in persistently positive cases, or monitoring for 2 years based on the CSO result. This approach could provide more reassurance to patients with a false positive MCED test result regarding their residual cancer risk, and could prevent unnecessary sequential referrals and associated delays in cancer diagnosis from falsely reassuring initial symptom-based assessment.^28^ Although a false positive MCED test result may lead to anxiety for patients, having a defined process to follow-up patients with a positive result, as described above, will help to reduce fear for patients who have a positive MCED test result and by providing reassurance to those who have a negative work-up along each step in the pathway.

There are different points along the diagnostic pathway where an MCED test could inform clinical decision making and referrals. To realise the potential benefits of the MCED test outlined in this manuscript, the MCED test would be requested at the time of the urgent suspected cancer referral and the test result returned prior to the cancer investigations indicated by the symptoms. The clinical team could choose to change the symptom directed investigations to investigations indicated by the CSO call or add further investigations to the symptom directed investigations based on the CSO call. To realise the additional potential benefits of identifying patients for urgent cancer referral not currently referred, and of reducing unnecessary urgent cancer referrals, the MCED test would need to be requested in primary care prior to referral and ideally at the time of the patient's initial symptomatic presentation to primary care. To avoid unnecessary MCED test use in patients who will already have a timely cancer diagnosis, the MCED test could be used solely in patients with negative cancer investigations and ongoing symptoms. However, this strategy would not reduce unnecessary urgent cancer referrals, identify additional patients who require urgent cancer referral, nor redirect urgent cancer investigations towards the correct cancer site.

For any strategy to be successful, the clinician should receive the MCED test result within a timeframe aligned to the clinical pathway. A thorough health economic analysis of each scenario would be required to balance the costs of MCED test use, diagnostic delay, and changes in investigative and referral behaviour.

### Conclusions

We found that only 252 (47%) cancers were diagnosed within 28 days of referral. We identified 101 (19%) cancers that may have been diagnosed more efficiently if the MCED test had been used in the diagnostic work-up of suspected cancers, and among these, 79 (78%) were diagnosed outside of the 28-day standard. If the MCED test would have been used in the diagnostic work-up of these patients, and if using the MCED test could have helped achieve the timelier diagnosis, this could have increased the percentage of cancers who were diagnosed within 1 month of urgent referral from 55% to 62%, indicating a substantial improvement in timelier diagnosis. We found that among the cancer sites that would have benefitted the most from the MCED test were hard to identify cancers, including cancers of unknown primary, gallbladder, pancreas, and ovarian. Many of these cancers are difficult to detect early and so using the MCED test in the diagnostic work-up may lead to invaluable improvements in time to diagnosis. For these benefits to be realised in clinical practice, the MCED test would most likely need to be returned to the requesting clinician in under two weeks.

## Contributors

AJ, BDN, MRM, KR, SH, HK, RP, and JLO designed the study. Data analysis was done by AJ and PSV. Supervision was provided by BDN and MRM. Data interpretation was done by AJ, PSV, ST, JLO, RP, FDRH, MRM, and BDN. Data are held by the academic partner and were accessed and verified by AJ, ST, and PSV. This report was initially drafted by AJ, BDN, and MRM and was reviewed and approved for publication by all co-authors, including those from the funder, and the sponsor. The authors vouch for the completeness and accuracy of the data and the data analyses. All authors had full access to all data in the study and had final responsibility for the decision to submit for publication.

## Data sharing statement

De-identified individual-level patient data can be provided to researchers upon written request 24 months after publication of the Article. Please send enquiries to the corresponding author. A detailed proposal for how the data will be used is required to allow assessment of the application.

## Declaration of interests

BDN and MRM receive institutional research funding from GRAIL for the SYMPLIFY study. HK, SH, and KR are/were employees of GRAIL at the time of the study, and KR and SH have stocks associated with their employment with GRAIL. HK reports a leadership position with GRAIL as president. All other authors declare no competing interests.

## Appendix A Supplementary data

Supplementary Tables

Protocol

Supplementary Material

Additional Follow Up Registry

## References

[1] Ahmad A.S., Ormiston-Smith N., Sasieni P.D. (2015). Trends in the lifetime risk of developing cancer in Great Britain: comparison of risk for those born from 1930 to 1960. Br J Cancer.

[2] NHS England (2022).

[3] Crosby D., Bhatia S., Brindle K.M. (2022). Early detection of cancer. Science.

[4] NHS England (2022).

[5] NHS England Earlier and faster diagnosis. https://www.england.nhs.uk/cancer/quarterly-report-overviews/q4-2021-q1-2122/earlier-and-faster-diagnosis/#:%7E:text=The%20Faster%20Diagnosis%20Standard%20is,quarters%20(75%25)%20of%20patients.

[6] Jensen H., Tørring M.L., Olesen F., Overgaard J., Vedsted P. (2014). Cancer suspicion in general practice, urgent referral and time to diagnosis: a population-based GP survey and registry study. BMC Cancer.

[7] Erridge S., Lyratzopoulos G., Renzi C., Millar A., Lee R. (2021). Rapid Diagnostic Centres and early cancer diagnosis. Br J Gen Pract.

[8] Scott S.E., Gildea C., Nicholson B.D. (2023). Future cancer risk after urgent suspected cancer referral in England when cancer is not found: a national cohort study. Lancet Oncol.

[9] Nielsen N., Vedsted P., Jensen H. (2018). Risk of cancer and repeated urgent referral after negative investigation for cancer. Fam Pract.

[10] Bronkhorst A.J., Ungerer V., Holdenrieder S. (2019). Early detection of cancer using circulating tumor DNA: biological, physiological and analytical considerations. Crit Rev Clin Lab Sci.

[11] Crosby D. (2022). Delivering on the promise of early detection with liquid biopsies. Br J Cancer.

[12] Schrag D., Beer T.M., McDonnell C.H. (2023). Blood-based tests for multicancer early detection (PATHFINDER): a prospective cohort study. Lancet.

[13] Park J.H., Oh Y., Chung L.I.-Y. (2023). Systematic review and meta-analysis of the accuracy and applicability of blood-based multi-cancer early detection (MCED) in the general population. J Clin Oncol.

[14] Lennon A.M., Buchanan A.H., Kinde I. (2020). Feasibility of blood testing combined with PET-CT to screen for cancer and guide intervention. Science.

[15] Schmeising-Barnes N., Waller J., Marlow L.A.V. (2024). Attitudes to multi-cancer early detection (MCED) blood tests for population-based screening: a qualitative study in Great Britain. Soc Sci Med.

[16] Jiang H., Gus W. (2024). Multi-cancer early detection tests: pioneering a revolution in cancer screening. Clinical Cancer Bulletin.

[17] Nicholson B.D., Oke J., Virdee P.S. (2023). Multi-cancer early detection test in symptomatic patients referred for cancer investigation in England and Wales (SYMPLIFY): a large-scale, observational cohort study. Lancet Oncol.

[18] Jackson A., Virdee P.S., Tonner S. (2024). Validity and timeliness of cancer diagnosis data collected during a prospective cohort study and reported by the English and Welsh cancer registries: a retrospective, comparative analysis. Lancet Oncol.

[19] Næser E., Fredberg U., Møller H., Vedsted P. (2017). Clinical characteristics and risk of serious disease in patients referred to a diagnostic centre: a cohort study. Cancer Epidemiol.

[20] Chapman D., Poirier V., Vulkan D., ACE MDC projects (2020). First results from five multidisciplinary diagnostic centre (MDC) projects for non-specific but concerning symptoms, possibly indicative of cancer. Br J Cancer.

[21] Dolly S.O., Jones G., Allchorne P. (2021). The effectiveness of the Guy's Rapid Diagnostic Clinic (RDC) in detecting cancer and serious conditions in vague symptom patients. Br J Cancer.

[22] Sewell B., Jones M., Gray H. (2020). Rapid cancer diagnosis for patients with vague symptoms: a cost-effectiveness study. Br J Gen Pract.

[23] Westgate C., Gordon O., Margolis M. (2024). 1184P early real-world experience with positive multi-cancer early detection (MCED) test cases and negative initial diagnostic work-up. Ann Oncol.

[24] Black G.B., Boswell L., Harris J., Whitaker K.L. (2023). What causes delays in diagnosing blood cancers? A rapid review of the evidence. Prim Health Care Res Dev.

[25] Lyratzopoulos G., Vedsted P., Singh H. (2015). Understanding missed opportunities for more timely diagnosis of cancer in symptomatic patients after presentation. Br J Cancer.

[26] Nicholson B.D., Hamilton W., Koshiaris C., Oke J.L., Hobbs F.D.R., Aveyard P. (2020). The association between unexpected weight loss and cancer diagnosis in primary care: a matched cohort analysis of 65,000 presentations. Br J Cancer.

[27] Zakkak N., Barclay M.E., Swann R. (2024). The presenting symptom signatures of incident cancer: evidence from the English 2018 National Cancer Diagnosis Audit. Br J Cancer.

[28] Roberts K., Cooper N., Webster L. (2025). Characterising the volume and variation of multiple urgent suspected cancer referrals in England, April 2013-March 2018: a national cohort study. BMJ Open.

