# Supplementary material for: Diagnostic Implications of Multi-Cancer Early Detection Testing in the Investigation of Cancer Symptoms: An Exploratory Retrospective Analysis of the SYMPLIFY Study
Source: Lancet Reg Health Eur. 2026 May 28;66:101720. doi: 10.1016/j.lanepe.2026.101720 (PMC13235408; doi:10.1016/j.lanepe.2026.101720)
Supplement: Supplementary Tables [file mmc1.pdf]

Supplementary Appendix

Supplement to: The potential diagnostic efficiency of adding MCED testing to the investigation of cancer symptoms: an exploratory study based on a two-year follow-up of SYMPLIFY study participants

TABLE OF CONTENTS

Tables 3

Table S1. Characteristics of cancers diagnosed among participants initially thought to be False Positive and True Negative in the initial SYMPLIFY study................. 3-4

Table S2. Line listing of patients initially deemed False Positive in original SYMPLIFY study who were later found to have cancer within 24 months.............................5

Tables

Supplementary Table 1. Characteristics of cancers diagnosed among participants initially thought to be False Positive and True Negative in the initial SYMPLIFY study.

Characteristic	False Positive (N=31)		True Negative (N=115)	
	Number (%)	Median days to diagnosis (IQR)	Number (%)	Median days to diagnosis (IQR)
**Whole Cohort**	31 (100)	120 (35-460)	115 (100)	405 (147-583)
**Age**				
50-59	4 (13)	263.5 (12-615)	21 (18)	467 (172-536)
60-69	7 (23)	114 (21-335)	23 (20)	406 (147-578)
70-79	9 (29)	156 (35-362)	46 (40)	365.5 (172-599)
80-89	5 (16)	392 (49-423)	18 (16)	370 (143-599)
90+	4 (13)	516 (224-668)	-	-
<50	2 (6)	93 (91-95)	7 (6)	330 (3-540)
**Sex**				
Female	17 (55)	119 (68-392)	65 (57)	355 (121-568)
Male	14 (45)	241 (21-548)	60 (52)	453 (167-605)
**Cancer Stage**				
Stage I	5 (16)	95 (0-503)	57 (50)	406 (147-561)
Stage II	6 (19)	93.5 (21-380)	11 (10)	491 (195-546)
Stage III	6 (19)	289.5 (120-553)	21 (18)	336 (95-583)
Stage IV	12 (39)	102.5 (29.5-397.5)	8 (7)	623.5 (514.5-676)
Unknown	2 (6)	377 (362-392)	18 (16)	219 (81-405)
**Cancer Site**				
Bladder and urothelial tract	-	-	12 (10)	283 (44.5-540)
Breast, female	-	-	21 (18)	491 (406-68)
CNS	-	-	1 (1)	405 (-)
Cervix	-	-	1 (1)	668 (-)
Colorectal	7 (23)	91 (49-460)	9 (8)	499 (272-616)
Head and neck	1 (3)	35 (-)	-	-
Liver, bile duct	2 (6)	457.5 (362-553)	2 (2)	24.5 (2-47)
Lung, trachea, and bronchus	8 (26)	199.5 (19.5-484.5)	15 (13)	502 (172-599)
Lymphoid	1 (3)	19 (-)	7 (6)	219 (143-509)

Melanoma of skin	1 (3)	119 (-)	7 (6)	317 (29-482)
Myeloid	-	-	1 (1)	391 (-)
Oesophagus	2 (6)	470 (392-548)	4 (3)	73 (30-208)
Other	1 (3)	21 (-)	7 (6)	90 (70-690)
Ovarian	4 (13)	156.5 (135-330)	1 (1)	189 (-)
Pancreas	1 (3)	15 (-)	4 (3)	114.5 (42.5-342)
Plasma cell	2 (6)	423.5 (120-727)	3 (3)	210 (195-585)
Prostate	-	-	16 (14)	481.5 (311-636)
Thyroid	-	-	1 (1)	495 (-)
Uterus	1 (3)	95 (-)	3 (3)	262 (0-624)
**Referral Pathway**				
Gynae 2WW	5 (16)	156 (119-157)	21 (18)	537 (254-599)
Lower GI clinic	9 (29)	460 (95-652)	47 (41)	273 (121-605)
Lung 2WW	6 (19)	22.5 (15-548)	7 (6)	391 (8-624)
Rapid diagnostic centre	3 (10)	362 (120-392)	10 (9)	301.5 (90-467)
Upper GI 2WW	8 (26)	66 (42-213)	30 (26)	451.5 (190-540)
**Time to Diagnosis**				
Within 28 days	7 (23)	15 (0-21)	11 (10)	2 (0-9)
>28 days	24 (77)	348.5 (104.5-524.5)	104 (90)	451.5 (202.5-589.5)

Supplementary Table 2. Line listing of patients initially deemed False Positive in original SYMPLIFY study who were later found to have cancer within 24 months.

Participant	Cancer Site	Stage	MCED CSO 1	MCED CSO 2	Congruency	Time to Diagnosis (days)
**1**	Ovarian	stage 4	ovary	uterus	Symptoms, pathway, MCED	157
**2**	Ovarian	stage 1	ovary	uterus	Symptoms, pathway, MCED	503
**3**	Uterus	stage 1	uterus	NA	MCED only	95
**4**	Lung, trachea, bronchus	stage 4	lung	lung/NET	Symptoms, MCED	64
**5**	Pancreas	stage 4	pancreas/gallbladder	NA	Symptoms, MCED	15
**6**	Colorectal	stage 4	colorectal	NA	Symptoms, pathway, MCED	460
**7**	Liver, Bile Duct	stage 3	liver/bile duct	NA	MCED only	553
**8**	Head and neck	stage 4	head and neck	lung	MCED only	35
**9**	Plasma Cell	stage 2	plasma cell	sarcoma	MCED only	727
**10**	Ovarian	stage 3	uterus	breast	Symptoms, pathway	156
**11**	Colorectal	stage 4	colorectal	NA	Symptoms, MCED	91
**12**	Colorectal	stage 2	colorectal	NA	Symptoms, MCED	380
**13**	Lung, trachea, bronchus	stage 2	lung	NA	Symptoms, pathway, MCED	15
**14**	Oesophagus	stage 4	upper GI	NA	MCED only	548
**15**	Other	stage 2	lung	head and neck	Symptoms, pathway, MCED	21
**16**	Colorectal	stage 1	colorectal	prostate	Symptoms, pathway, MCED	0
**17**	Lung, trachea, bronchus	stage 1	lung	NA	Symptoms, pathway, MCED	0
	Lung, trachea, bronchus	stage 4	lung	NA	Symptoms, pathway, MCED	24
**18**	Colorectal	stage 2	lymphoid	colorectal	None	68
**19**	Lymphoid	stage 4	lymphoid	NA	MCED only	19
**20**	Oesophagus	unknown	colorectal	upper GI	Symptoms, pathway	392
**21**	Plasma Cell	stage 3	plasma cell	liver/bile duct	Symptoms, pathway, MCED	120
**22**	Colorectal	stage 3	colorectal	NA	Symptoms, MCED	49
**23**	Ovarian	stage 4	ovary	uterus	Symptoms, pathway, MCED	114
	Melanoma of skin	stage 2	ovary	uterus	None	119
**24**	Lung, trachea, bronchus	stage 4	lung	NA	MCED only	546
**25**	Colorectal	stage 3	colorectal	NA	Symptoms, pathway, MCED	652
	Lung, trachea, bronchus	stage 1	colorectal	NA	None	684
**26**	Lung, trachea, bronchus	stage 4	lung/NET	lung	Symptoms, MCED	335
**27**	Liver, Bile Duct	unknown	lung	upper GI	Symptoms, pathway	362
**28**	Lung, trachea, bronchus	stage 3	head and neck	lung	None	423
