# Supplementary material for: Diagnostic Implications of Multi-Cancer Early Detection Testing in the Investigation of Cancer Symptoms: An Exploratory Retrospective Analysis of the SYMPLIFY Study
Source: Lancet Reg Health Eur. 2026 May 28;66:101720. doi: 10.1016/j.lanepe.2026.101720 (PMC13235408; doi:10.1016/j.lanepe.2026.101720)
Supplement: Protocol [file mmc2.pdf]

Study Title: SYMPLIFY – Observational study to assess a multi-cancer early detection test in individuals referred with signs and symptoms of cancer

Internal Reference Number / Short title: SYMPLIFY

Ethics Ref: 21/LO/0456

IRAS Project ID: 299310

Date and Version No: v6.0 17 Dec 2024

**Chief Investigator:**	Mark R. Middleton, PhD, FRCP<br>Professor of Experimental Cancer Medicine<br>University of Oxford Department of Oncology<br>Old Road Campus Research Building<br>Roosevelt Drive<br>Oxford, OX3 7DQ<br>Tel: +44 (0)1865 617331<br>Email: mark.middleton@oncology.ox.ac.uk
**Lead Investigator:**	Brian D Nicholson, MRCGP, DPhil<br>NIHR Academic Clinical Lecturer<br>Nuffield Department of Primary Health Care Sciences<br>Radcliffe Primary Care Building<br>Radcliffe Observatory Quarter<br>Woodstock Road, Oxford. OX2 6GG<br>Tel: +44 (0)1865 289300<br>Email: brian.nicholson@phc.ox.ac.uk
**Sponsor:**	Research Governance, Ethics & Assurance Team<br>Joint Research Office,<br>University of Oxford,<br>Boundary Brook House<br>Churchill Drive, Headington<br>Oxford OX3 7GB<br>Tel: 01865 616480<br>E-mail: rgea.sponsor@admin.ox.ac.uk
**Chief Investigator Signature:**	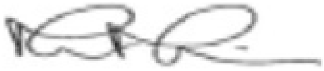

No conflicts of interest are declared by the trial team

Confidentiality Statement

© Copyright: The University of Oxford and Oxford University Hospitals NHS Foundation Trust 2019

This document contains confidential information that must not be disclosed to anyone other than the Sponsor, the Investigator Team, HRA, host organisation, and members of the Research Ethics Committee, unless authorised to do so.

© Copyright: The University of Oxford and Oxford University Hospitals NHS Foundation Trust 2019

TABLE OF CONTENTS

1. KEY CONTACTS 5
2. LAY SUMMARY 6
3. SYNOPSIS 7
4. ABBREVIATIONS 9
5. BACKGROUND AND RATIONALE 10
5.1. Background 10
5.2. GRAIL's MCED test 11
6. OBJECTIVES AND OUTCOME MEASURES 12
7. STUDY DESIGN 13
8. PARTICIPANT IDENTIFICATION 14
8.1. Study Participants 14
8.2. Inclusion Criteria 14
8.3. Exclusion Criteria 14
9. PROTOCOL PROCEDURES 15
9.1. Recruitment 15
9.2. Screening and Eligibility Assessment 15
9.3. Informed Consent 15
9.4. Registration 16
9.5. Description of study procedure 16
9.6. Study Visit 16
9.7. Follow-ups 16
9.8. Early Discontinuation/Withdrawal of Participants 17
9.9. Definition of End of Study 17
10. SAFETY REPORTING 17
11. STATISTICS AND ANALYSIS 17
11.1. Statistical Analysis Plan (SAP) 17
11.2. Analysis populations 18
11.3. Data collected 18
11.4. Description of the Statistical Methods 18
11.5. Sample Size Determination 20
12. DATA MANAGEMENT 20
12.1. Source Data 20
12.2. Electronic Data Capture 21
12.3. Access to Data 21
12.4. Data Recording and Record Keeping 21
13. QUALITY ASSURANCE PROCEDURES 21

© Copyright: The University of Oxford and Oxford University Hospitals NHS Foundation Trust 2019

13.1. Risk assessment 22
13.2. Study monitoring 22
13.3. Study Committees 22
14. PROTOCOL DEVIATIONS 22
15. SERIOUS BREACHES 22
16. ETHICAL AND REGULATORY CONSIDERATIONS 22
16.1. Declaration of Helsinki 22
16.2. Guidelines for Good Clinical Practice 22
16.3. Approvals 23
16.4. Other Ethical Considerations 23
16.5. Reporting 23
16.6. Transparency in Research 23
16.7. Participant Confidentiality 23
16.8. Expenses and Benefits 24
17. SPONSORSHIP, FINANCE AND INSURANCE 24
17.1. Sponsorship 24
17.2. Funding 24
17.3. Insurance 24
17.4. Contractual arrangements 24
18. PUBLICATION POLICY 24
19. DEVELOPMENT OF A NEW PRODUCT/ PROCESS OR THE GENERATION OF INTELLECTUAL PROPERTY 25
20. RECORD RETENTION & ARCHIVING 25
21. REFERENCES 26
● APPENDIX A: AMENDMENT HISTORY 27

© Copyright: The University of Oxford and Oxford University Hospitals NHS Foundation Trust 2019

1. KEY CONTACTS

**Chief Investigator**	Mark R. Middleton, PhD, FRCP<br>Professor of Experimental Cancer Medicine<br>University of Oxford Department of Oncology<br>Old Road Campus Research Building<br>Roosevelt Drive<br>Oxford, OX3 7DQ<br>Tel: +44 (0)1865 617331<br>Email: mark.middleton@oncology.ox.ac.uk
**Sponsor**	Research Governance, Ethics & Assurance Team<br>Joint Research Office,<br>University of Oxford,<br>Boundary Brook House<br>Churchill Drive, Headington<br>Oxford OX3 7GB<br>Tel: 01865 616480<br>E-mail: rgea.sponsor@admin.ox.ac.uk
**Funder(s)**	Funded by an unrestricted educational grant from GRAIL Bio UK Ltd. Supported by NHS England, NHS Wales, the National Institute for Health Research (NIHR) and the Oxford NIHR Biomedical Research Centre.
**Clinical Trials Unit(s)**	SYMPLIFY Study Team<br>Oncology Clinical Trials Office (OCTO)<br>Department of Oncology<br>Old Road Campus Research Building<br>Old Road Campus<br>Roosevelt Drive<br>Headington<br>Oxford OX3 7DQ<br>Email: octo-symplify@oncology.ox.ac.uk<br><br>Primary Care & Vaccine Collaborative Clinical Trials Unit<br>Nuffield Department of Primary Care Health Sciences,<br>Gibson Building, 1st Floor, Radcliffe Observatory Quarter,<br>Oxford OX2 6GG<br>Email: primarycarectu@phc.ox.ac.uk
**Statistician**	Rafael Perera Salazar<br>Professor of Medical Statistics<br>Nuffield Department of Primary Health Care Sciences<br>Radcliffe Primary Care Building<br>Radcliffe Observatory Quarter<br>Woodstock Road, Oxford. OX2 6GG<br>rafael.perera@phc.ox.ac.uk<br>Tel: 01865 289308<br>Fax: 01865 289287
**Committees**	No study specific monitoring committee will be engaged.<br><br>The Trial Management Group (TMG) will be responsible for day to day conduct of the study. The TMG will consist of the Chief (Chair) and Lead Investigators, Trials Unit representatives, a Sponsor representative and representatives from GRAIL.

© Copyright: The University of Oxford and Oxford University Hospitals NHS Foundation Trust 2019

	The study will be overseen by the relevant oversight committees of the two trials units involved, taking into account input from the GRAIL Clinical Advisory Group

2. LAY SUMMARY

Cancer is easier to treat if it is diagnosed early. The NHS has developed rapid referral pathways to allow cancers to be diagnosed earlier, but this requires us to run tests on a lot more people than turn out to have cancers. This creates worry for some people, may not be the most efficient use of NHS resources, a significant proportion of cancers are diagnosed via other routes, often when it is too late to cure them. We know that the DNA from cancers can be detected in the blood early on and want to use this fact to detect cancers earlier than existing pathways. In this study we want to check the performance of one such *multi-cancer early detection* (MCED) test. We will do this in people sent to one of 5 rapid referral pathways by their GP because they have symptoms that might be due to cancer. People taking part in the study will have their diagnostic test(s) in the normal way, but will also give a blood sample and permission for us to check their health records later to see if they were diagnosed with cancer and what appointments and other tests they had. At the end of the study, having tested the blood with the MCED test, we will understand more about how well it works in this group of people, and expect that this will help us to design another trial where we check how to use the test to decide who needs rapid referral to look for a possible cancer and what tests to use following a positive MCED result

© Copyright: The University of Oxford and Oxford University Hospitals NHS Foundation Trust 2019

3. SYNOPSIS

Study Title	SYMPLIFY - Observational study to assess a multi-cancer early detection test in individuals referred with signs and symptoms of cancer
Internal ref. no. / short title	SYMPLIFY<br>(Grail-010)
Sponsor	University of Oxford Research Governance, Ethics & Assurance Team<br>Joint Research Office,<br>University of Oxford,<br>Boundary Brook House<br>Churchill Drive, Headington<br>Oxford OX3 7GB Tel: 01865 616480<br>E-mail: rgea.sponsor@admin.ox.ac.uk
Funder	Funded by an unrestricted educational grant from GRAIL Bio UK Ltd. Supported by NHS England, NHS Wales, the National Institute for Health Research (NIHR) and the Oxford NIHR Biomedical Research Centre.
Study Design	Prospective observational multi-centre cohort
Study Participants	Adults aged 18yrs + referred to NHS urgent 2WW and RDC cancer pathways
Sample Size	6000 (approx. 500: lung 2WW; 1000: gynae 2WW; 2000: upper GI 2WW; 2000: lower GI 2WW; 500: RDC)
Planned Study Period	01 Jun 2021 to 31 January 2026
Planned Recruitment period	01 Jul 2021 to 30 Sept 2021
Intervention	This is not an interventional study

**Objectives**	**Outcome Measures**	**Timepoint(s) of evaluation of this outcome measure**
**Primary Objective**		
To evaluate the performance of a MCED test for the detection of invasive cancer	Positive Predictive Value<br>Negative Predictive Value<br>Sensitivity<br>Specificity	An interim analysis within 3 months of complete enrolment with complete analysis at 12 months of enrolment.
**Secondary Objectives**		
To evaluate the performance of a MCED test by referral pathway (i.e. lung, upper GI, lower GI, gynae, and RDC) and cancer type and stage	Positive Predictive Value<br>Negative Predictive Value<br>Sensitivity<br>Specificity	An interim analysis within 3 months of complete enrolment with complete analysis at 12 months of enrolment.
To evaluate the performance of a MCED test for the identification of cancer signal origin	Cancer signal origin accuracy, overall by cancer site and by pathway selected by the GP	An interim analysis within 6 months of complete enrolment with complete analysis at 12 months of enrolment.
To evaluate the yield with MCED by referral pathway	Number of true positives/number of patients referred within each referral pathway	An interim analysis within 3 months of complete enrolment

© Copyright: The University of Oxford and Oxford University Hospitals NHS Foundation Trust 2019

		with complete analysis at 12 months of enrolment.
**Exploratory Objectives**		
To evaluate the completeness of patient data collected from central databases	Proportion of completed data fields, according to locally and centrally sourced inputs	Locally within 3 months and by 9 months of enrolment, and centrally monthly from 3 through 12 months post enrolment.
To investigate if clinical parameters further optimise the performance of MCED test	Positive Predictive Value<br>Negative Predictive Value<br>Sensitivity<br>Specificity	At 12 months post enrolment
To evaluate the time to diagnostic resolution by referral pathway	Days between enrolment and diagnostic resolution	At 12 months post enrolment
To estimate resource utilisation by referral pathway	Number of encounters, tests, and referrals required to achieve diagnostic resolution	At 12 months post enrolment
To evaluate the yield of non-cancer diagnoses following referral.	Number of patients diagnosed with non-cancer/number of patients referred	At 12 months post enrolment
To investigate cancers diagnosed within 2 years of a false positive MCED result	Cancer number, timing, site, morphology, and stage	Recorded in central cancer registry within 24 months of enrolment

© Copyright: The University of Oxford and Oxford University Hospitals NHS Foundation Trust 2019

4. ABBREVIATIONS

2WW	Two-Week-Wait
CI	Chief Investigator
CRF	Case Report Form
CSO	Cancer Signal Origin
GCP	Good Clinical Practice
GP	General Practitioner
HRA	Health Research Authority
ICF	Informed Consent Form
MCED	Multi-Cancer Early Detection
NHS	National Health Service
RDC	Rapid Diagnostic Centre
RES	Research Ethics Service
RGEA	Research Governance, Ethics & Assurance, University of Oxford (Sponsor)
PI	Principal Investigator
PIL	Participant/ Patient Information Leaflet
R&D	NHS Trust R&D Department
REC	Research Ethics Committee
SOP	Standard Operating Procedure

© Copyright: The University of Oxford and Oxford University Hospitals NHS Foundation Trust 2019

5. BACKGROUND AND RATIONALE

5.1. Background

Delays in cancer diagnosis can impact both disease outcome and patient and provider experience. Detection of cancer and subsequent intervention at earlier stages of disease may greatly improve patient outcomes and reduce overall cancer-related mortality. Earlier intervention has been shown to improve patient outcomes, including overall survival (World Health Organization Guide to Cancer: Early Diagnosis. 2017). Results from a nationwide Danish study (Robinson 2012) evaluating the association between diagnostic delay, quality of life and patient satisfaction, showed reduced quality of life and patient satisfaction with diagnostic delay for ovarian and endometrial cancer. Expediting symptomatic diagnosis for cancers can be achieved by having a high index of suspicion for cancer when the patient first contacts the healthcare system, the early use of appropriate diagnostic technologies, and access to fast-track pathways for assessment.

Cancer screening is only routinely available for breast, colorectal, and cervical cancers in England and Wales, identifying <10% of adult cancers. Nine out of ten people diagnosed with cancer visit their GP with symptoms in the year prior to being diagnosed (Hamilton et al). GPs urgently refer patients with symptoms and signs of cancer according (in England) to a set of nationally agreed NICE guideline criteria, based on these symptoms, via two-week-wait (2WW) pathways for a specialist appointment within that time (Thompson, M/CADEAS). A core ambition within the NHS Long Term Plan, is to diagnose 75% of cancers at an early stage by 2028, and in previous iterations, has been to further lower the referral threshold to a cancer risk of ≤3% (NHS Long Term Plan). Patients diagnosed with earlier stage cancer have improved outcomes due to the increased opportunity for use of potentially curative treatments, which are often not an option or are less effective for later stage cancers (Miller et al). For example, studies in pancreatic cancer indicate that the diagnostic interval can affect the odds of upfront surgical resection and thus the chance of potentially curative surgery in symptomatic patients (Deshwar).

The number and proportion of cancers diagnosed via 2WW pathways has steadily increased over time (Zhou, Y). By 2020, 53.5% of all cancers were diagnosed following 2WW referral. However, unsurprisingly, over the same time, the 2WW conversion rate (the proportion of people referred to 2WW who are diagnosed with cancer through that referral) has fallen from one in ten referred patients (10.8% in 2009/10) to one in fifteen (6.6% in 2019/20). With increasing referral rates and decreasing conversion rates, hospitals are struggling to cope with an unsustainable increase in demand for radiological and endoscopic procedures (Overview | Suspected cancer: recognition and referral | Guidance | NICE). Due to the limited predictive value of symptoms, patients can require a number of 2WW referrals to different specialties before a cancer diagnosis is reached. It is clear that additional parameters are needed to assist GP decision-making, both in terms of whether a 2WW referral is required and to direct to the most appropriate organ-specific clinic.

Non-2WW GP urgent or routine referrals make up the second most common route to a cancer diagnosis in the NHS after 2WW. Non-2WW pathways include multiple referral pathways into numerous clinical specialities and have low and highly variable cancer conversion rates (<1%). Our study, which is delivered in secondary care, therefore focuses on 2WW referral pathways and Rapid Diagnostic Centres (RDCs).

RDCs (Rapid Diagnostic Centres: Vision and 2019/20 Implementation Specification), previously Multidisciplinary Diagnostic Centres (MDCs), are being set-up at pace across the NHS to diagnose patients with non-specific symptoms (weight loss, fatigue, appetite loss, abdominal pain) who do not meet 2WW

© Copyright: The University of Oxford and Oxford University Hospitals NHS Foundation Trust 2019

criteria. Patients referred to RDC/MDC pathways are rapidly and broadly investigated in order to reduce delays caused by these patients being referred to multiple 2WW and non-2WW pathways in sequence until a diagnosis is reached. Whilst the risk of individual cancers is low in the RDC/MDC pathways, the combined risk for cancer of any type is high, with conversion rates higher than most 2WW pathways. Across the five MDC pilots in England, 241 cancers were diagnosed following 2961 referrals, with a conversion rate of 8.1% spread across multiple cancer sites. Of those cancers with stage data available, 25 (13.0%) were Stage I, 24 (12.4%) Stage II, 39 (20.2%) Stage III, and 105 (54.4%) Stage IV. RDCs, however, are resource intensive: for example, all patients referred to some RDCs undergo full body low-dose Computed Tomography (CT), blood tests, specialist review, with onward radiological and endoscopic investigations as indicated. A multi-cancer early detection test (MCED) that predicts the tissue of origin could both help establish cancer as a likely source of symptoms and direct further evaluation to the predicted tissue of origin.

5.2. GRAIL's MCED test

GRAIL's Galleri™ MCED blood test is a qualitative, next-generation sequencing (NGS)-based screening test using cell-free DNA isolated from adult human peripheral whole blood. When a cancer signal is detected, the test can also localise the cancer signal with high accuracy. The test report describes one or two Cancer Signal Origins (CSOs). If the first CSO score is high (≥9.0), then only one CSOs is reported, otherwise the two top CSOs are reported. MCED has 21 possible CSOs: anus; bladder; urothelial tract; bone and soft tissue; breast; cervix; colon, rectum; head and neck; kidney; liver/bile duct; lung; lymphoid lineage; melanocytic lineage; myeloid lineage; neuroendocrine; ovary; pancreas, gallbladder; plasma cell lineage; prostate; stomach, oesophagus; thyroid gland; uterus. It is designed as a screening test and not to confirm a cancer diagnosis. Although the test result of "cancer signal detected" with Cancer Signal Origin may indicate the presence of cancer, further investigations to diagnose cancer are necessary in accordance with professional guidelines.

Preliminary evaluations of the GRAIL approach to blood markers were presented in 2018 and 2019. The Circulating Cancer Genome Atlas (CCGA) study (Clinicaltrials.gov identifier: NCT02889978) enrolled ~10,000 participants with cancer and 5,000 participants without a clinical diagnosis of cancer from medical institutions and networks throughout the United States and Canada. The pre-specified locked assay and classifier that was developed, analytically validated, and clinically validated in the CCGA study will be used in this study. The investigational test system includes bioinformatic analysis pipeline software that uses the targeted methylation assay output to detect cancer and predict CSO, blood collection tubes (cell-free DNA blood collection tubes manufactured by Streck, Inc.), GRAIL's study test kit, and an NGS assay.

The first CCGA classification analysis (CCGA1) consisted of 2,800 participants, including 1,650 participants with newly diagnosed cancer who had not yet received treatment and 1,150 non-cancer participants. The prototype assays detected a highly specific, strong biological signal in cancer types that are typically not screened for and have low survival rates (five-year cancer-specific mortality rate of greater than 50 percent): lung, ovarian, pancreatic, liver, and oesophageal cancers. For these cancers in the CCGA1 Training set at 98% specificity, the sensitivity for stages I-III cancers (N=117) was 54% with the highest performing prototype assay, whole genome bisulfite sequencing. Sensitivity increased to 90% for stage IV cancers (N=81). Longitudinal follow-up has confirmed that three of eight participants who had an elevated signal but no cancer diagnosed at the time were later diagnosed with cancer suggesting that the signal indicated the presence of cancer before it was detectable by diagnostics used in clinical practice.

© Copyright: The University of Oxford and Oxford University Hospitals NHS Foundation Trust 2019

The second CCGA cohort (CCGA2) included ~4,800 participants and used a targeted methylation (TMe) cfDNA assay. This cohort served as the basis for the development of the GRAIL test classifier that distinguishes cancer from non-cancer and identifies cancer signal origin. CCGA2 included individuals presenting to clinical attention prior to cancer diagnosis with signs and/or symptoms ultimately resulting in a diagnosis of cancer. Non-cancer participants were defined by having no cancer at enrolment and non-cancer status confirmed at year one follow-up. This study demonstrated the ability of the test to achieve high specificity (99.5%, 95%CI 98.2-99.9%) in the non-cancer group, moderate sensitivity in those presenting to clinical attention (66.4%, 95%CI 62.2-70.3%), and high overall accuracy of cancer signal origin prediction (91.7%, 95% CI: 88.3-94.3%) in those presenting to clinical attention.

Within this context, GRAIL’s MCED blood test could complement the existing 2WW and RDC pathways by offering a novel approach to 2WW and non-2WW pathway selection for people presenting to their GP with symptoms that could be from cancer. Here, we evaluate the performance of the test in predicting cancer versus non-cancer as well as cancer signal origin for multiple cancers in secondary care cancer clinics. This includes cancers with high 5-year mortality that do not currently have a screening programme and some for which workup based on symptoms is not yet standardised. Impact on management, time to diagnosis and resource utilisation are assessed within the NHS context.

GRAIL's Galleri^{TM} MCED blood test is not currently CE (UKCA) marked.

This study is classified as a Performance Evaluation of an In-Vitro Diagnostic Device (PEIVDD). The test (device) will be registered with the MHRA as an IVD undergoing Performance Evaluation. The study will not open to recruitment until this registration is confirmed.

6. OBJECTIVES AND OUTCOME MEASURES

Objectives	Outcome Measures	Timepoint(s) of evaluation of this outcome measure
**Primary Objective**		
To evaluate the performance of a MCED test for the detection of invasive cancer	Positive Predictive Value<br>Negative Predictive Value<br>Sensitivity<br>Specificity	An interim analysis within 3 months of complete enrolment with complete analysis at 12 months of enrolment.
**Secondary Objectives**		
To evaluate the performance of a MCED test for the detection of invasive cancer by referral pathway (i.e. lung, upper GI, lower GI, gynae, and RDC) and cancer type and stage	Positive Predictive Value<br>Negative Predictive Value<br>Sensitivity<br>Specificity	An interim analysis within 3 months of complete enrolment with complete analysis at 12 months of enrolment.
To evaluate the performance of a MCED test for the identification of cancer signal origin	Cancer signal origin accuracy, overall by cancer site and by pathway selected by the GP	An interim analysis within 6 months* of complete enrolment with complete analysis at 12 months of enrolment.
To evaluate the yield with MCED by referral pathway	Number of true positives/number of patients referred within each referral pathway	An interim analysis within 3 months of complete enrolment with complete analysis at 12 months of enrolment.
**Exploratory Objectives**		

© Copyright: The University of Oxford and Oxford University Hospitals NHS Foundation Trust 2019

To evaluate the completeness of patient data collected from central databases	Proportion of completed data fields, according to locally and centrally sourced inputs	Locally within 3 months and by 9 months of enrolment, and centrally monthly from 3 through 12 months post enrolment.
To investigate if clinical parameters further optimise the performance of MCED test	Positive Predictive Value<br>Negative Predictive Value<br>Sensitivity<br>Specificity	At 12 months post enrolment
To evaluate the time to diagnostic resolution by referral pathway	Days between enrolment and diagnostic resolution	At 12 months post enrolment
To estimate resource utilisation by referral pathway	Number of encounters, tests, and referrals required to achieve diagnostic resolution	At 12 months post enrolment
To evaluate the yield of non-cancer diagnoses following referral.	Number of patients diagnosed with non-cancer/number of patients referred	At 12 months post enrolment
To investigate cancers diagnosed within 2 years of a false positive MCED result	Cancer number, timing, site, morphology, and stage	Recorded in central cancer registry within 24 months of enrolment

* the 6 month CSO analysis will use the same locked dataset as the 3 month interim analysis. The 3-6 month period is to allow CSO mapping to be conducted by GRAIL and the CSO analysis conducted by the University, using the 3 month data after the 3 month dataset is locked by the University and the test results have been received from GRAIL and locked by the University.

7. STUDY DESIGN

This is a multi-centre, observational study with prospective collection and retrospective analysis of blood samples to evaluate the performance of a multi-cancer early detection test within the NHS in England and Wales.

The study will enrol 6000 participants over a period of approximately 3 months. Individuals who have been referred to a 2WW or RDC urgent cancer pathway by their GP will be invited to take part. Recruitment will take place in secondary care following referral, when patients attend for investigation. The anticipated breakdown by pathway is as follows:

- **lung 2WW** (n=500, expected cancer conversion rate = 16%); the majority of cancers diagnosed are expected to be lung cancer
- **gynae 2WW** (n=1000, expected cancer conversion rate = 4%); the majority of cancers diagnosed are expected to be ovarian cancer
- **upper GI 2WW** (n=2000, expected cancer conversion rate = 4%); the majority of cancers diagnosed are expected to be oesophagus, stomach, pancreas or stomach cancers,
- **lower GI clinic** (n=2000, expected cancer conversion rate = 4%); the majority of cancers diagnosed are expected to be colorectal cancer
- **Rapid Diagnostic Centres** (RDC) (n=500, expected cancer conversion rate = 7%); the majority of cancers diagnosed are expected to be pancreatic, other upper GI, lung, colorectal, lymphoma, multiple myeloma, and renal tract.

© Copyright: The University of Oxford and Oxford University Hospitals NHS Foundation Trust 2019

Following informed consent, participants will be registered and assigned a study participant identification number. Approximately 40 mL of whole peripheral blood will be collected from all participants. The blood samples collected from participants will be shipped to a laboratory in the UK for processing to plasma and storage. As the MCED test will not be run until after the patient completes their investigative pathway, as the test requires clinical validation, no individual results will be returned to the study participants or the clinicians responsible for their care.

Clinical information and demographic data will be collected from all participants via case report forms and NHS datasets. There are no protocol-required diagnostic procedures. Decisions regarding which specific diagnostic procedures should be performed and all medical decision making will be according to established clinical practice in the relevant clinical pathway.

The result of any diagnostic procedure(s) will be recorded until the date of diagnostic resolution, with patient level data collected from both participating hospital records and NHS datasets. Data will be collected within 3 months of enrolment to identify cancer diagnoses and serious disease outcomes, and at a later single time point between 6 and 9 months of enrolment for those without diagnostic resolution at 3 months. At 12 and 24 months, patient level data will be collected from primary and secondary care electronic health records and linked NHS datasets to capture cancers diagnosed later and healthcare utilisation following recruitment. Participants will therefore be involved only at their initial visit.

Diagnostic resolution outcomes that will be confirmed by clinical review of cases where there is uncertainty will include:

- **Cancer diagnosis**: pathologic confirmation of an invasive or haematologic malignancy, or other accepted criteria in the absence of pathology.
- **Non-cancer diagnosis**: diagnosis other than cancer at the conclusion of diagnostic evaluation for the presenting complaint.
- **No diagnosis**: no diagnosis of cancer and no non-cancer condition diagnosed to explain the presenting complaint.

The primary analysis will occur when all data is collected and the database locked. An interim analysis will occur within 3 months of the enrolment of all participants.

8. PARTICIPANT IDENTIFICATION

8.1. Study Participants

Participants referred to an RDC or relevant 2WW pathway to rule a cancer diagnosis in or out will be invited to participate in the study.

8.2. Inclusion Criteria

- Willing and able to give informed consent for participation in the study.
- Male or Female, aged 18 years or above.
- Referred to a RDC or a gynae, lung, upper GI or lower GI cancer 2WW pathway.

8.3. Exclusion Criteria

A potential participant may not enter the study if ANY of the following apply:

- has a history of invasive or haematological malignancy diagnosed within the previous 3 years

© Copyright: The University of Oxford and Oxford University Hospitals NHS Foundation Trust 2019

- has undergone definitive treatment for invasive or haematological malignancy in the last 3 years (adjuvant hormone therapy is permissible in this context).
- is taking cytotoxic or demethylating agents such as methotrexate
- previous or current participation in another GRAIL study. "Participation" is defined as having signed consent and provided a blood sample.

9. PROTOCOL PROCEDURES

9.1. Recruitment

Recruitment will take place at secondary care sites in the selected English Cancer Alliance and Welsh Health Board territories. Sites with high volumes for the included 2WW pathways and/or active RDCs will be selected.

All patients referred to the four 2WW pathways or RDC will be invited to participate. Written patient information will be provided at first attendance, or may be provided to patients in advance where local arrangements for the pathway permit.

9.2. Screening and Eligibility Assessment

No exceptions will be made regarding eligibility: each participant must satisfy all the approved inclusion and exclusion criteria of the protocol. Screening for prior cancer will be undertaken by questioning potential participants. Rescreening is not applicable to the study.

9.3. Informed Consent

The participant must personally sign and date the latest approved version of the Informed Consent Form before any study specific procedures is performed.

Written and verbal versions of the Participant Information and Informed Consent will be presented to potential participants detailing the nature of the study and what it will involve for the participant. It will be clearly stated that the participant is free to withdraw from the study at any time for any reason without prejudice to future care, without affecting their legal rights, and with no obligation to give the reason for withdrawal. Where possible, information may be shared with potential participants prior to their first clinical visit. Sites may communicate study information via the channels they routinely use to communicate information related to their 2WW or RDC appointment. However, as the 2WW and RDC pathways are designed to provide rapid cancer investigation, time may not permit an approach before patients attend for investigation.

In such circumstances the potential participant will be given time to consider the study information and the opportunity to ask questions but will need to decide whether they will participate in the study at their first clinical visit. Although unusual, the lack of time for reflection before providing consent is mitigated by the right to withdraw subsequently and by the very limited requirements of participation (a single blood draw). Written Informed Consent will then be obtained by means of participant-dated signature and dated signature of the person who presented and obtained the Informed Consent. The person who obtained the consent must be suitably qualified and experienced and have been authorised to do so by the Principal Investigator. A copy of the signed Informed Consent will be given to the participant. The original signed form will be retained at the study site. A further copy will accompany a letter to a participant's GP, if it is necessary to obtain information about the participant from their records.

The Consent Form will contain a discreet clause that addresses the use of remaining samples for further exploratory research beyond the scope of this protocol. This research may include additional biological

© Copyright: The University of Oxford and Oxford University Hospitals NHS Foundation Trust 2019

analysis of samples and the development of new methods to analyse these biological data. Further research may be used to further develop GRAIL products and the MCED test. The further use of samples will not be specifically optional and participants will be told that they are free to refuse to participate and may withdraw their consent at any time and for any reason during the period of the SYMPLIFY study, per section 9.8.

9.4. Registration

Participants will be registered immediately after providing informed consent. This will generate a unique participant identification number used to label and track blood samples drawn for the study.

Patient registration will be via a secure online system: https://sentry.phc.ox.ac.uk/sentry/

9.5. Description of study procedure

Participants will undergo a single blood draw of ~40mL at the study visit. Blood will be collected ideally from a single venepuncture site into Streck™ tubes. Where possible blood should be drawn at the same time as routine venepuncture or cannulation is taking place, but a study specific draw is permitted. Please refer to the study specific sample handling manual for detailed instructions on blood collection, sample labelling and shipping.

9.6. Study Visit

At the **first 2WW or RDC appointment**, staff will collect baseline data from all consented participants using a paper-based or electronic case report form (CRF). Paper-based approaches will be retained to acknowledge that NHS England infrastructure is not yet reliable in all corners of all NHS Trusts. All paper-based CRF data will be uploaded by the study team onto the secure online OpenClinica research database. eCRF data will automatically populate the OpenClinica database.

Approximately 40 mL of whole venous blood will be taken from all participants using aseptic technique into Streck™ CE cfDNA BCT tubes, and packaged in the consumables for shipping processing and storage. Samples will be sent to a central sample handling facility for processing in the UK. Processing will create isolated plasma which will be sent to the USA for analysis in batches. Sample identification numbers will be logged on the participant CRF.

9.7. Follow-ups

By **three months** of follow-up, research staff will complete follow-up CRFs after reviewing the participant's hospital records. The research staff will review the hospital record again by **nine months** of follow-up for those participants for whom diagnostic resolution is not reached by the three months of follow-up. This additional follow-up is required to ensure cancer stage information is complete, as for many participants this will not be adequately recorded at 3 months of follow-up or in the cancer registry linked at 12 and 24 months. CRF data will be compared with the same data captured centrally from NHS datasets.

At **twelve months** of follow-up, CRF data will be linked to primary and secondary care electronic health records data to allow validation of eCRF data and cancer outcomes data, and the capture of primary and secondary care activity data from before and after enrolment. Data from before enrolment will allow a more detailed characterisation of participants, such as previous diagnoses and comorbidities, their contacts with primary care and secondary care in the year prior to enrolment, and tests performed in primary care leading up to the date of referral. Our experience tells us that, to ensure a complete dataset, PC-CTU trial administrators and research nurses will be required to review the linked data retrieved for each participant, and then work with a subset of GP practices for which the linked data is incomplete. Due

© Copyright: The University of Oxford and Oxford University Hospitals NHS Foundation Trust 2019

to the 18-month validation process for the full National Cancer Registrations Analysis Service (NCRAS) cancer registry, we will also link to the cancer outcomes and services dataset (COSD) data and NCRAS rapid cancer registrations dataset. Equivalent datasets will be accessed for participants recruited in Wales. Primary care clinical and activity data will be retrieved by accessing the Summary Care Record where possible, if this is unavailable for any reason the participant's GP will be contacted for the same or equivalent information. For imaging activity in primary and secondary care we will access the diagnostic imaging dataset (DID). To capture secondary care activity, we will link to hospital event statistics (HES) and secondary uses service (SUS) data.

At **twenty four** months of follow up CRF data will be linked to primary and secondary care electronic health records data to capture any further diagnoses of cancer in participants with a false positive MCED result.

9.8. Early Discontinuation/Withdrawal of Participants

During the course of the study a participant may choose to withdraw early. Participants may withdraw their consent, meaning that they wish to withdraw from the study completely. At this point it should be ascertained if participants wish to EITHER

- withdraw from the study but permit data and samples obtained up until the point of withdrawal to be retained for use in the study analysis. No further data would be collected after withdrawal OR
- withdraw completely from the study and withdraw the data and samples collected up until the point of withdrawal. The data and samples already collected would not be used in the final study analysis.

The type of withdrawal and reason for withdrawal will be recorded in the CRF. If a withdrawal occurs during the recruitment period, the patient will be replaced during the recruitment window. If the withdrawal occurs following the recruitment period they will not be replaced. Participants can request sample withdrawal until the end of the study only and will be made aware of this in the PIS.

9.9. Definition of End of Study

The end of study is the point at which all the final participant's last data has been captured.

10. SAFETY REPORTING

Safety reporting is not judged necessary for the study as patients undergo a single blood draw as the only study procedure. Where possible venepuncture will be done at the time of clinical blood draw to avoid an additional procedure. Blood sampling by venepuncture has a very well understood spectrum of adverse effects, being undertaken hundreds of thousands of times daily across the NHS.

11. STATISTICS AND ANALYSIS

We plan to enrol 6000 patients, comprising 500 from the lung 2WW pathway, 1000 from the gynae 2WW pathway, 2000 each from the upper and lower GI 2WW pathways and 500 referred to RDCs. Our targeted enrolment strategy is expected to yield a cancer conversion rate of ~5%.

11.1. Statistical Analysis Plan (SAP)

© Copyright: The University of Oxford and Oxford University Hospitals NHS Foundation Trust 2019

The statistical aspects of the study are summarised here with details fully described in a statistical analysis plan (SAP). The SAP will be developed and finalised before database lock and will describe the participant populations to be included in the analyses, and the procedures to address missing, unused, and spurious data.

11.2. Analysis populations

For the purposes of analysis, the following populations are defined:

- **Enrolled**: all consented participants
- **Clinically Evaluable**: consented participants who are clinically evaluable. Evaluability is defined as completing the standard investigative pathway to rule cancer in or out, with sufficient clinical data to assure the determination
- **Analysable**: all clinically evaluable participants with evaluable MCED test results
- **Diagnostic Resolution**: clinically evaluable patients with an analysable MCED test result whose diagnosis was resolved at the time of analysis.

11.3. Data collected

Categories of data collection will include the following:

- Participant related identifiers (e.g., NHS number, DOB)
- Demographics (e.g., ethnicity, sex)
- Baseline clinical information (e.g., smoking, alcohol use, personal and family history of cancer, questions on cancer screening adherence)
- Referral information: Type of referral clinic and pathway, referral criteria
- Cancer and other diagnosis information at follow up timepoints: diagnostic resolution, tests completed as part of diagnostic work up, cancer diagnosis and associated tumour characteristics, and staging.

11.4. Description of the Statistical Methods

Participant characteristics will be summarised using descriptive statistics with figures and/or tables as appropriate. These summaries will be stratified by the MCED test result, referral pathway, and cancer type and stage. For cancer types that have 10 or fewer observations, they will be grouped and summarized as "other."

Primary analysis

The primary analysis is to evaluate the performance (sensitivity, specificity, PPV and NPV) of the MCED test for the detection of newly-diagnosed invasive cancer based on cancer signal detected results of the MCED test. . Two separate sets of estimates of test performance for the MCED test will be obtained. An early interim estimate which will be based on data captured within 3 months after enrolment and a late/complete estimate based on data at 12 months after enrolment to account for delayed diagnoses (including the 9-month and 12-month follow-up data).

All results, including failures and equivocal results, will be reported using tables, while analysis of test performance will be based on valid results only (analysable population). The MCED test performance parameters PPV, NPV, sensitivity, specificity will be evaluated overall and reported using point estimates

© Copyright: The University of Oxford and Oxford University Hospitals NHS Foundation Trust 2019

along with 95% CIs. The PPV of the MCED test will be compared to the PPV (the “conversion rate”) of the included pathways. The interim analysis will estimate MCED test performance parameters based on the cancer site of the cancer pathway referral and not the cancers predicted by the MCED (as cancers predicted by MCED that do not align with the referral pathway chosen may be diagnosed later in the follow-up period). The interim analysis will summarise the status of the full cohort but the calculation of test performance will only include participants who have reached diagnostic resolution related to the initial cancer pathway referral. Two-sided 95% confidence intervals (CIs) will be constructed for proportion estimates using the Wilson (score) method (Wilson, 1927), unless otherwise specified. Tables and/or figures will be used to report these estimates with associated 95%CIs.

Secondary analyses

The first secondary analysis will stratify the performance (sensitivity, specificity, PPV and NPV) of the MCED test for the detection of invasive cancer by referral pathway (i.e. lung, upper GI, etc.) and by cancer type and clinical stage.

To meet our second secondary objective, a comparison will be made between a target signal origin label and the top-one predicted signal origin label from the MCED report. Three different/distinct target signal origin labels will be determined by (i) clinical outcome in NHS services, (ii) CSO mapping by clinical scientists at GRAIL, and (iii) the participant's general practitioner's (GP) choice of referral criteria.

For participants for whom the GP was unable to predict the cancer signal (e.g. by referring to an RDC pathway) or the MCED result was no cancer signal detected, their results will be included as separate categories in a separate column/row with the available target CSOs reported.

Yield will be estimated based on the number of cancer cases that were also MCED test positive (true positives) out of the total tested (i.e. analysable population). Yield estimates will be presented overall, and by referral pathway using point estimates and 95%CI.

Exploratory analyses

A key output for the study will be an analysis of the completeness and quality of cancer diagnostic pathway data gathered from central NHS databases. Datapoints collected locally within 3 months and by 9 months of enrolment will be compared to data collected centrally monthly from 3 through 12 months post enrolment. The completeness, by capture method, and concordance between methods of each data field will be summarised descriptively. This will inform the potential to use central data capture for future large-scale studies in the field, as well as identify areas for improvement in central data linkage.

We will also investigate if patient demographic characteristics, referral information, and other relevant clinical parameters allow for further optimisation of the performance of MCED test. The full dataset will be split into 70% for training and 30% as a testing set. Given the number of expected cancer events in the training set (6000*70%=4200 participants with a 5% estimated cancer rate = 210), a model taking into account all cancers would potentially allow us to explore between 10-21 variables including the MCED test (signal detected/not detected and predicted CSO from the test) (based on 20 or 10 events per variable in the model). To achieve this, we will use penalized regression models, such as lasso regression, aiming to reduce the amount of overfitting. The k-fold cross-validation will also be employed in the training set to fine-tune the tuning parameters and further reduce the amount of overfitting. The models developed from

© Copyright: The University of Oxford and Oxford University Hospitals NHS Foundation Trust 2019

the training set will be evaluated in the holdout test set. These models will be used as hypothesis generating only and will require validation using new and independent data.

The time from enrolment to diagnosis will be analysed overall and by referral pathway, using the survival methods (e.g. Kaplan-Meier method).

The following resource utilisation measurements will be summarized descriptively, overall and by referral pathway: number of encounters to diagnosis; number and types of tests for diagnosis; comparisons of resource utilisation observed to modelled resource utilisation based on cancer signal detected and CSO. These data will be used to evaluate the cost-effectiveness of the MCED test compared to standard care from an NHS perspective. Our analysis will determine the healthcare resources and costs required for the GRAIL pathway compared to the standard care pathways for these patient groups. This will include estimation of the costs of alternative diagnostic tests, primary care and secondary care visits and contacts. Our initial outcome measure would include simply cancer cases detected, with a cost-per-life-year-saved estimated once longer-term follow-up data are available. Finally, we will also undertake a budget impact analysis to help determine whether introducing the MCED test into routine care in this setting is likely to be affordable for the NHS.

Serious disease yield will also be estimated based on the number serious disease cases diagnosed out of the total enrolled.

We intend to follow-up all MCED results classified as false positive based on the data available from study sites using two years of follow- up in national cancer registry data. This analysis aims to understand whether there were cancers diagnosed later that were undetected at the time of initial investigation, whether these cancers were missed due to referrals initially being made to a cancer pathway that was incongruent with the eventual cancer diagnosis identified or whether no cancer was detectable by standard of care investigations conducted at the time of referral.

11.5. Sample Size Determination

The latest NHS England two week wait clinic data (from 2018) were used to estimate the distribution by cancer type and stage within each pathway. Sensitivity estimates by cancer type and stage for GRAIL v2.9 training and holdout, cross-validated data using isotonic regression were used at a specificity of 99.4% to estimate the expected PPV and NPV for a given sample size. It is expected that with a sample size of 6000 the PPV will be 86.8% (95% CI: 82.1%, 90.7%) and NPV will be 98.6% (95% CI: 98.3%, 98.9%). The anticipated contribution of each clinical pathway to the 6000 participants is discussed in section 7.

12. DATA MANAGEMENT

A summary plan for the data management of the study is outlined below.

12.1. Source Data

Source documents are where data are first recorded, and from which participants' CRF data are obtained. These include, but are not limited to, primary care and hospital records (from which medical history, diagnostic tests and primary and secondary care visits and contacts may be drawn into the CRF).

CRF entries will be considered source data if the CRF is the site of the original recording (e.g. there is no other written or electronic record of data). All documents will be stored safely in confidential conditions. On study-specific documents, other than the signed consent, the participant will be referred to by their

© Copyright: The University of Oxford and Oxford University Hospitals NHS Foundation Trust 2019

unique participant identification number/code, not by name. Patient identifiers (including NHS number) collected at baseline will be used for linkage to NHS datasets.

CRF data at baseline and months 3 and 9 will be entered by site staff from site records, including but not limited to individual patient records and 2WW tracking systems. 12-month and 24-month data will be extracted from primary care records, Hospital Episode Statistics, Cancer Outcomes and Services Dataset and Secondary Uses Service data.

The same datapoints for months 3, 9 and 12 will be populated independently from central datasets, including national cancer registry data. The accuracy and completeness of centrally collected data will be compared with that collected at the site for the baseline and within 3-month groups.

CRF data from 3 and 9 months will be used for the interim and final analyses, respectively. Before CSO mapping is performed, the Galleri™ MCED test results will be transferred from Grail and held in the trial database by the sponsor.

Data collected from central cancer registries at 24-months will be used to conduct an exploratory analysis on false positive MCED test results. Case reports forms (CRFs)

The Investigator and study site staff will ensure that data collected on each participant is recorded in the CRF as accurately and completely as possible at the relevant timepoints. All appropriate data, will be transcribed into the CRFs from the relevant source data held in the site medical record(s).

The above considerations also apply to patients who withdraw consent. If a patient withdraws from the study, the reason must be noted on the appropriate form and the SYMPLIFY study team at Oxford notified immediately.

12.2. Electronic Data Capture

Electronic data capture (EDC) and data management will be performed via a web-based, bespoke trial database (OpenClinica). OpenClinica is a dedicated and validated clinical trials database designed for electronic data capture. The trials office will provide sites with instructions and a link to online training.

https://phc.openclinica.io/

12.3. Access to Data

Direct access will be granted to authorised representatives from the Sponsor and host institution for monitoring and/or audit of the study to ensure compliance with regulations.

12.4. Data Recording and Record Keeping

All study data will be entered on Case Report Form (CRFs) at site at baseline, and for 3 month and, where applicable, 9-month data capture. 12-month and 24-month data will be captured from primary and secondary care records. The same data will be collected in parallel from NHS databases centrally.

The participants will be identified by a unique study specific number in the study database.

Patient identifiers including initials, NHS number and year of birth will be retained centrally (i.e. by the CTU within the University of Oxford), for linkage with NHS datasets.

13. QUALITY ASSURANCE PROCEDURES

© Copyright: The University of Oxford and Oxford University Hospitals NHS Foundation Trust 2019

The study may be monitored, or audited in accordance with the current approved protocol, GCP, relevant regulations and standard operating procedures.

13.1. Risk assessment

A risk assessment and monitoring plan will be prepared before the study opens and will be reviewed as necessary over the course of the study to reflect significant changes to the protocol or outcomes of monitoring activities.

13.2. Study monitoring

No routine on-site monitoring will be undertaken, in keeping with an observational study with a single study procedure that does not impact clinical care. Locally collected and centrally extracted data will be compared to assess the relative merits of the two approaches for future studies, and to provide assurance of the quality of the study data. The CTUs will manage the data collection according to SOPs covering central monitoring, with development of central monitoring activities to ensure oversight of the sites and quality of the data entered.

13.3. Study Committees

No study specific monitoring committee will be engaged. The Trial Management Group (TMG) will be responsible for day to day conduct of the study. The TMG will consist of the Chief and Lead Investigators, Trials Unit representatives, a Sponsor representative and representatives from GRAIL. The study will be overseen by the relevant oversight committees of the two trials units involved, taking into account input from the GRAIL Clinical Advisory Group.

14. PROTOCOL DEVIATIONS

Protocol compliance is fundamental to GCP. Changes to the approved protocol need prior approval unless for urgent safety reasons. The investigator must document and explain any deviations/violations from the current approved protocol. The investigator must promptly report any important deviation from Good Clinical Practice or protocol to the study office by email. Examples of important deviations are those that might impact on patient safety, primary/ secondary endpoint data integrity, or be a possible serious breach of GCP.

15. SERIOUS BREACHES

A “serious breach” is a breach of the protocol or of the conditions or principles of Good Clinical Practice which is likely to affect to a significant degree –

(a) the safety or physical or mental integrity of the study subjects; or

(b) the scientific value of the research.

Investigators must notify the Trials Office immediately if any serious breach is suspected. In the event that a serious breach is suspected the Sponsor must be contacted within 1 working day. In collaboration with the C.I., the serious breach will be reviewed by the Sponsor and, if appropriate, the Sponsor will report it to the approving REC committee and the relevant NHS host organisation within seven calendar days.

16. ETHICAL AND REGULATORY CONSIDERATIONS

16.1. Declaration of Helsinki

The Sponsor and Investigators will ensure that this study is conducted in accordance with the ethical principles of the Declaration of Helsinki and applicable policies of the sponsor.

16.2. Guidelines for Good Clinical Practice

© Copyright: The University of Oxford and Oxford University Hospitals NHS Foundation Trust 2019

The Sponsor and Investigators will ensure that this study is conducted in accordance with relevant regulations and with Good Clinical Practice.

16.3. Approvals

Following Sponsor approval, the protocol, informed consent form, participant information sheet and any proposed additional material will be submitted to an appropriate Research Ethics Committee (REC), and HRA and host institutions for written approval.

The sponsor will submit and, where necessary, obtain approval from the above parties for all substantial amendments to the original approved documents.

16.4. Other Ethical Considerations

MCED test results will not be available in real time and will not be reported to study participants nor to site investigators. Participants will undergo standard investigations to rule cancer in or out according to established practice, so the test will not impact on their care.

16.5. Reporting

The CI shall submit once a year throughout the study, or on request, an Annual Progress Report to the REC Committee, HRA host organisation, Sponsor and funders. In addition, an End of Study notification and final report will be submitted to the same parties.

16.6. Transparency in Research

Prior to the recruitment of the first participant, the study will have been registered on a publicly accessible database.

Where the study has been registered on multiple public platforms, the study information will be kept up to date during the study, and the CI or their delegate will upload results to all those public registries within 12 months of the end of the study declaration.

16.7. Participant Confidentiality

The study will comply with the UK General Data Protection Regulation (GDPR) and Data Protection Act 2018, which require data to be anonymised as soon as it is practical to do so. Personal data recorded on all documents will be regarded as confidential. The processing of the personal data of participants will be minimised by making use of a unique participant identification number only on all study documents and any electronic database(s), with the exception of the CRF.

The patient's name (on the consent form only), NHS number, GP Practice details, date of birth, postcode and sex will be collected once to allow flagging with NHS England

Participant initials and NHS number do not form part of the research data for analysis. All documents will be stored securely and only accessible by study staff and authorised personnel. The study staff will safeguard the privacy of participants' personal data.

Data Breaches will be highlighted to the relevant site staff and reported as required by the UK GDPR and Data Protection Act 2018. This will also be deemed a protocol deviation.

The Investigator site must maintain the patient's anonymity in all communications and reports related to the research. The Investigator site team must keep a separate log of enrolled patients' personal identification details as necessary to enable them to be tracked. These documents must be retained securely, in strict confidence. They form part of the Investigator Site File and are not to be released externally. Data Breaches

© Copyright: The University of Oxford and Oxford University Hospitals NHS Foundation Trust 2019

will be highlighted to the relevant site staff and reported as required by the UK GDPR and Data Protection Act 2018. This will also be deemed a protocol deviation.

Participants have consented for the study team to follow up their medical records for 12 months after study entry. In order to follow up their medical records for an additional 12 months (24 months in total) we will seek 251 exemption from the Confidentiality Advisory Group (CAG). Participants will be notified by letter of our intention for additional data collection and provided with contact details to allow them opt out if they wish.

16.8. Expenses and Benefits

As the study visit will take place when participants attend secondary care for their standard investigations, no travel expenses will be reimbursed.

17. SPONSORSHIP, FINANCE AND INSURANCE

17.1. Sponsorship

The Sponsor will provide written confirmation of Sponsorship. The CTU study teams will authorise the study commencement once satisfied that all arrangements and approvals for the proper conduct of the study are in place.

17.2. Funding

The study is funded by an unrestricted educational grant from GRAIL, who will also be coordinating the shipping of blood samples from NHS sites, processing blood samples obtained from participants into isolated plasma, and undertaking and reporting the MCED test.

In kind support is provided by NHS England and NHS Wales to interrogate central datasets and to assist with project management. The National Institute for Health Research (NIHR) is making staff available across its Clinical Research Network to assist with patient recruitment and site level data capture. The Oxford NIHR Biomedical Research Centre provides core resource to the Oncology Clinical Trials Office and funds the Chief Investigator.

17.3. Insurance

The University has a specialist insurance policy in place which would operate in the event of any participant suffering harm as a result of their involvement in the research (Newline Underwriting Management Ltd, at Lloyd's of London). NHS indemnity operates in respect of the clinical procedure undertaken.

17.4. Contractual arrangements

This study is subject to the Sponsor's policy requiring that written contracts/agreements are agreed formally by the participating bodies as appropriate.

The Sponsor will also set up written agreements with any other external third parties involved in the conduct of the study as appropriate.

The division of responsibilities for each group within the sponsor organisation will be documented in the Trial Master File.

18. PUBLICATION POLICY

The Investigators will be involved in reviewing drafts of the manuscripts, abstracts, press releases and any other publications arising from the study. Authors will acknowledge that the study was funded by GRAIL with in kind funding from NHS England. Authorship will be determined in accordance with the ICMJE guidelines and other contributors will be acknowledged.

© Copyright: The University of Oxford and Oxford University Hospitals NHS Foundation Trust 2019

19. DEVELOPMENT OF A NEW PRODUCT/ PROCESS OR THE GENERATION OF INTELLECTUAL PROPERTY

Ownership of IP generated by employees of the University vests in the University. The University will ensure appropriate arrangements are in place as regards any new IP arising from the study.

20. RECORD RETENTION & ARCHIVING

During the study and after study closure the Investigator must maintain adequate and accurate records to enable the conduct of a study and the quality of the research data to be evaluated and verified. All essential documents must be stored in such a way that ensures that they are readily available, upon request for the minimum period required by national legislation or for longer if needed. The medical files of study participants must be retained in accordance with applicable national legislation and the host institution policy.

Retention and storage of laboratory records for clinical study samples must also follow these guidelines.

Retention and storage of central laboratory records and the disposition of samples donated via the study must also comply with applicable legislation and Sponsor requirements.

It is the University of Oxford's policy to store data for a minimum of 3 years from publication. Investigators may not archive or destroy study essential documents or samples without written instruction from the study office.

Study data and associated metadata will be retained electronically in a suitable format in a secure server area maintained and backed up to the required standard. Access will be restricted to the responsible Archivist and will be controlled by a formal access request. On completion of the mandatory archiving period the study master file and associated archived data sets will be destroyed or transferred as appropriate, according to any data sharing requirements.

© Copyright: The University of Oxford and Oxford University Hospitals NHS Foundation Trust 2019

21. REFERENCES

CADEAS. Available at: http://www.ncin.org.uk/local_cancer_intelligence/cadeas#covid-19. (Accessed: 29th January 2021)

Deshwar AB et al. Diagnostic intervals and pancreatic ductal adenocarcinoma (PDAC) resectability: a single-center retrospective analysis; Ann Pancreat Cancer. **1**, 13. doi: 10.21037/apc.2018.02.01 (Feb 2018)

Hamilton W, Walter F M, Rubin G & Neal RD Improving early diagnosis of symptomatic cancer. *Nature Reviews Clinical Oncology* **13**, 740–749 (2016).

Miller KD, et al. Cancer Treatment and Survivorship Statistics, 2016. CA Cancer J Clin. **66**, 271-289 (2016).

NHS Long Term Plan. Available at: https://www.longtermplan.nhs.uk/. (Accessed: 29th January 2021)

Overview | Suspected cancer: recognition and referral | Guidance | NICE.

Rapid Diagnostic Centres: Vision and 2019/20 Implementation Specification.

Robinson K et al. Diagnostic delay, quality of life and patient satisfaction among women diagnosed with endometrial or ovarian cancer: a nationwide Danish study. Quality of Life Research, November 2012, Volume 21, Issue 9 https://link.springer.com/article/10.1007%2Fs11136-011-0077-3

Thompson M et al. Have large increases in fast track referrals improved bowel cancer outcomes in UK? BMJ **371**, (2020).

World Health Organization. (2017). Guide to cancer early diagnosis. World Health Organization. https://apps.who.int/iris/handle/10665/254500.

Zhou Y et al. Variation in ‘fast-track’ referrals for suspected cancer by patient characteristic and cancer diagnosis: Evidence from 670 000 patients with cancers of 35 different sites. Br J Cancer **118**, 24–31 (2018).

© Copyright: The University of Oxford and Oxford University Hospitals NHS Foundation Trust 2019

- **APPENDIX A: AMENDMENT HISTORY**

Amendment No.	Protocol Version No.	Date issued	Author(s) of changes	Details of Changes made
SA01	From 1.0 to 2.0	V1.0 20May2021	Brian Nicholson	Clarification of CSO accuracy assessment as a secondary objective. Update to name of the University sponsor office from CTRG to RGEA
NSA05	From 2.0 to 3.0	V2.0 19Jan2022	Sharon Tonner	Update of study end date to 31 January 2024 and update to University sponsor office email address.
SA02	From 3.0 to 4.0	V3.0 29 June 2023	Sharon Tonner	Update of study end date to 31 January 2025, addition of exploratory outcome - to investigate cancers diagnosed within 2 years of a false positive MCED result.
SA03	From 4.0 to 5.0	V4.0 29 January 2024	Sharon Tonner	NHS Digital changed to NHS England. Section 251 exemption from CAG to allow for additional follow up period added to section 16.7.
SA04	From 5.0 to 6.0	V5.0 09 April 2024	Sharon Tonner	Study end date extended to January 2026

© Copyright: The University of Oxford and Oxford University Hospitals NHS Foundation Trust 2019
