# Supplementary material for: Diagnostic Implications of Multi-Cancer Early Detection Testing in the Investigation of Cancer Symptoms: An Exploratory Retrospective Analysis of the SYMPLIFY Study
Source: Lancet Reg Health Eur. 2026 May 28;66:101720. doi: 10.1016/j.lanepe.2026.101720 (PMC13235408; doi:10.1016/j.lanepe.2026.101720)
Supplement: Supplementary Material [file mmc3.pdf]

STATISTICAL ANALYSIS PLAN

SYMPLIFY

Study Title: SYMPLIFY – Observational study to assess a multi-cancer early detection test in individuals referred with signs and symptoms of cancer

Internal Reference Number / Short title: SYMPLIFY

Ethics Ref: 21/LO/0456

IRAS Project ID: 299310

Date and Version No: v1.0 24 June 2022

Version History

Version:	Version Date:	Changes:
SYMPLIFY_SAP_v0.1_25Jun2021	25 June 2021	First version
SYMPLIFY_SAP_v0.2_17Jan2021	17 January 2022	Updated primary analysis, edited secondary analysis, edited exploratory analysis, added sensitivity analyses.
SYMPLIFY_SAP_v0.3_13Apr2022	13 April 2022	Included definition of 'confirmed' and 'probable' cancers<br>Added detail to secondary analysis.<br>Added appendices with information regarding secondary analysis<br>Added sensitivity analysis including only 'confirmed cancers'
SYMPLIFY_SAP_v0.4_6May2022	6 May 2022	Added categories for the secondary analyses to take into account no-cancers and other cancers detected based on NHS outcomes<br>Modified secondary analysis based on GP pathways to be based on a stratified analysis.<br>Added appendix 5 which includes templates of tables for CSO reporting.

SYMPLIFY_SAP_v0.5_26May 2022	26 May 2022	Addition of sensitivity analysis for the second secondary analysis based only on those that have a positive cancer diagnosis by the appropriate reference standard AND a positive cancer signal detected in the results of the MCED test. Clarification of Appendix 2.
SYMPLIFY_SAP_v0.6_13June 2022	13 June 2022	Change to text to reflect GRAIL's GalleriTM MCED blood test now currently CE and UKCA marked as a screening test in an asymptomatic population. Added exploratory analysis of MCED test compared to GP suspected cancer site. Addition of a third type of sensitivity analysis for the first secondary analysis based on cancer type as determined by GRAIL (see Appendix 7), instead of the NCRAS-RTD classification. Addition of Appendix 7.
SYMPLIFY_SAP_v1.0_24June 2022	24 June 2022	Finalized SAP. up-versioned to v1.0. Added SAP signature page.

The signature below constitutes the approval of this SAP.

DocuSigned by:
Mark R. Middleton
Signer Name: Mark R. Middleton
Signing Reason: I approve this document
Signing Time: 25-Jun-2022 | 1:52:02 PM PDT
4FF0650197034DED951DDDE22E9B9879

25-Jun-2022

Mark R. Middleton, PhD, FRCP — Date
Chief Investigator, University of Oxford

DocuSigned by:
Brian Nicholson
Signer Name: Brian Nicholson
Signing Reason: I approve this document
Signing Time: 29-Jun-2022 | 12:36:30 AM PDT
3F1166EF40F84B3FA4CF89125548FE35

29-Jun-2022

Dr Brian D Nicholson MRCGP DPhil — Date
Lead Investigator, University of Oxford

DocuSigned by:
Signer Name: Rafael Perera-Salazar
Signing Reason: I am the author of this document
Signing Time: 29-Jun-2022 | 2:17:03 AM PDT
5B83E4B504BC4C11B0CBD49AC01BA7C2

29-Jun-2022

Rafael Perera-Salazar — Date
Professor of Medical Statistics, University of Oxford

DocuSigned by:
Harpal Kumar
Signer Name: Harpal Kumar
Signing Reason: I approve this document
Signing Time: 26-Jun-2022 | 1:37:41 AM PDT
675B3B515E5543DBB6F874726AF4EACC

26-Jun-2022

Sir Harpal Kumar — Date
President, GRAIL Europe

DocuSigned by:
Elizabeth Holmes
Signer Name: Elizabeth Holmes
Signing Reason: I approve this document
Signing Time: 29-Jun-2022 | 4:32:49 AM PDT
A9A02571D9274B87A6D888CC8612AC87

29-Jun-2022

Dr. Elizabeth Holmes — Date
Senior Medical Director, GRAIL

DocuSigned by:
Kathryn Kurtzman
Signer Name: Kathryn Kurtzman
Signing Reason: I approve this document
Signing Time: 29-Jun-2022 | 5:18:26 AM PDT
3A80931F23774431B2CBDA842742C387

29-Jun-2022

Kathryn Kurtzman, MD — Date
Senior Medical Director, GRAIL

DocuSigned by:
Ying Luan
Signer Name: Ying Luan
Signing Reason: I approve this document
Signing Time: 27-Jun-2022 | 11:02:07 AM PDT
6F4FA7F072464A2BBB964B9284D8C5A3

27-Jun-2022

Ying Luan — Date
Director Biostatistics, GRAIL

DocuSigned by:
Nan Zhang
Signer Name: Nan Zhang
Signing Reason: I approve this document
Signing Time: 25-Jun-2022 | 7:37:22 PM PDT
ACBE55ADA0DE4C249B2DD6E8EB4C6103

25-Jun-2022

Nan Zhang, PhD — Date
Vice President Biostatistics, GRAIL

- TABLE OF CONTENTS

1 Introduction 6

1.1 Key contacts 6

1.2 Purpose and scope of the plan 7

1.3 Trial overview 7

1.3.1 Background 7

1.3.2 GRAIL's MCED test 8

1.4 Objectives 10

2 Study Methods 10

2.1 Study design 10

2.2 Index Test 11

2.3 Reference – Diagnostic Resolution 11

2.4 Order of testing and independence of results 12

2.5 Outcomes measures 12

2.5.1 Primary outcome 12

2.5.2 Secondary outcomes 12

2.5.3 Exploratory outcomes 12

2.6 Target population 13

2.6.1 Study Participants 13

2.6.2 Inclusion Criteria 13

2.6.3 Exclusion Criteria 13

2.7 Sample size 13

3 Analysis – General considerations 14

3.1 Descriptive statistics 14

3.2 Definition of population for analysis 14

3.3 Pooling of sites and referral pathways 14

3.4 Data Monitoring Committee And Interim Analyses 14

4 PRIMARY ANALYSIS 15

4.1 Primary outcome 15

4.2 Handling missing data 16

4.3 Handling multi-centre/clustered data 16

4.4 Multiple comparisons and multiplicity 16

4.5 Model assumptions 16

5 SECONDARY ANALYSIS 16

6 EXPLORATORY ANALYSIS 19
7 SENSITIVITY ANALYSIS 21
8 SUBGROUP ANALYSES 21
9 ADDITIONAL EXPLORATORY ANALYSIS 21
10 SAFETY ANALYSIS 21
10.1 Adverse events 21
11 VALIDATION 21
12 CHANGES TO THE PROTOCOL OR PREVIOUS VERSIONS OF SAP 22
13 References 24
APPENDIX 1. List of Signal Origin Labels 25
APPENDIX 2. Anticipated cancer types based on NCRAS-RTD classification 26
APPENDIX 3. List of Clinical Outcome in NHS Services based on NCRAS-RTD classification 27
APPENDIX 4: Signal Origins and associated subcategories 29
APPENDIX 5: Shell table to present MCED CSD and CSO results vs NHS outcome for all participants recruited. This table could be replicated for each clinical pathway. 30
APPENDIX 6: Shell table to present MCED CSD and CSO results vs CSO mapping by clinical scientists at GRAIL for all participants recruited. This table could be replicated for each clinical pathway. 31
APPENDIX 7 Anticipated cancer types based on GRAIL classification 32

1 INTRODUCTION

1.1 KEY CONTACTS

**Chief Investigator**	Mark R. Middleton, PhD, FRCP<br>Professor of Experimental Cancer Medicine<br>University of Oxford Department of Oncology<br>Old Road Campus Research Building<br>Roosevelt Drive<br>Oxford, OX3 7DQ<br>Tel: +44 (0)1865 617331<br>Email: mark.middleton@oncology.ox.ac.uk
**Lead Investigator**	Brian D Nicholson, DPhil, MRCGP<br>NIHR Academic Clinical Lecturer<br>Nuffield Department of Primary Care Health Sciences<br>Radcliffe Primary Care Building<br>Radcliffe Observatory Quarter<br>Woodstock Road, Oxford. OX2 6GG<br>Email: brian.nicholson@phc.ox.ac.uk
**Sponsor**	Clinical Trial and Research Governance Team<br>Joint Research Office,<br>University of Oxford,<br>1st floor, Boundary Brook House<br>Churchill Drive, Headington<br>Oxford OX3 7GB<br>Tel: 01865 616480<br>E-mail: ctrg@admin.ox.ac.uk
**Funder(s)**	Funded by an unrestricted educational grant from GRAIL Bio UK Ltd. Supported by NHS England, NHS Wales, the National Institute for Health Research (NIHR) and the Oxford NIHR Biomedical Research Centre.
**Clinical Trials Unit(s)**	SYMPLIFY Study Team<br>Oncology Clinical Trials Office (OCTO)<br>Department of Oncology<br>Old Road Campus Research Building<br>Old Road Campus<br>Roosevelt Drive<br>Headington<br>Oxford OX3 7DQ<br>Email: octo-symplify@oncology.ox.ac.uk<br><br>Primary Care & Vaccine Collaborative Clinical Trials Unit<br>Nuffield Department of Primary Care Health Sciences,<br>Gibson Building, 1st Floor, Radcliffe Observatory Quarter,<br>Oxford OX2 6GG<br>Email: primarycarectu@phc.ox.ac.uk
**Senior Statistician**	Rafael Perera Salazar<br>Professor of Medical Statistics

	Nuffield Department of Primary Health Care Sciences<br>Radcliffe Primary Care Building<br>Radcliffe Observatory Quarter<br>Woodstock Road, Oxford. OX2 6GG<br>rafael.perera@phc.ox.ac.uk<br>Tel: 01865 289308<br>Fax: 01865 289287
**Committees**	No study specific monitoring committee will be engaged.<br><br>The Trial Management Group (TMG) will be responsible for day to day conduct of the study. The TMG will consist of the Chief (Chair) and Lead Investigators, Trials Unit representatives, a Sponsor representative and representatives from GRAIL.<br>The study will be overseen by the relevant oversight committees of the two trials units involved, taking into account input from the GRAIL Clinical Advisory Group
**Study Statistician**	Jason Oke
**Trial Manager**	Clare Freestone
**Data Manager**	Daniel Plotkin

This document has been written based on information contained in the protocol version/name: SYMPLIFY_Protocol_v2_19 Jan 2022 .

1.2 PURPOSE AND SCOPE OF THE PLAN

This document provides an overview of the study, a description of the study methods, and the analysis plan for all outcomes including sensitivity analysis. The purpose of this document is to provide an agreed plan for the analysis of the study which meet the aims and objectives of the SYMPLIFY study
This document should be used in conjunction with all case report forms (CRFs) and the latest version of the Study Protocol.

1.3 TRIAL OVERVIEW

1.3.1 BACKGROUND

Detection of cancer and subsequent intervention at earlier stages of disease may greatly improve patient outcomes and reduce overall cancer-related mortality. Earlier intervention has been shown to improve patient outcomes, including overall survival (World Health Organization Guide to Cancer: Early Diagnosis. 2017). Expediting symptomatic diagnosis for cancers can be achieved by having a high index of suspicion for cancer when the patient first contacts the healthcare system, the early use of appropriate diagnostic technologies, and access to fast-track pathways for assessment.

GPs urgently refer patients with symptoms and signs of cancer according (in England) to a set of nationally agreed NICE guideline criteria, based on these symptoms, via two-week-wait (2WW) pathways for a specialist appointment within that time (Thompson, M/CADEAS). Patients diagnosed with earlier stage cancer have improved outcomes due to the increased

opportunity for use of potentially curative treatments, which are often not an option or are less effective for later stage cancers (Miller et al).

The number and proportion of cancers diagnosed via 2WW pathways has steadily increased over time (Zhou, Y). However, unsurprisingly, over the same time, the 2WW conversion rate (the proportion of people referred to 2WW who are diagnosed with cancer through that referral) has fallen from one in ten referred patients (10.8% in 2009/10) to one in fifteen (6.6% in 2019/20). Due to the limited predictive value of symptoms, patients can require a number of 2WW referrals to different specialties before a cancer diagnosis is reached. It is clear that additional parameters are needed to assist GP decision-making, both in terms of whether a 2WW referral is required and to direct to the most appropriate organ-specific clinic.

Rapid Diagnostic Centres (RDCs) (Rapid Diagnostic Centres: Vision and 2019/20 Implementation Specification), previously Multidisciplinary Diagnostic Centres (MDCs), are being set-up at pace across the NHS to diagnose patients with non-specific symptoms (weight loss, fatigue, appetite loss, abdominal pain) who do not meet 2WW criteria. Patients referred to RDC/MDC pathways are rapidly and broadly investigated in order to reduce delays caused by these patients being referred to multiple 2WW and non-2WW pathways in sequence until a diagnosis is reached. Whilst the risk of individual cancers is low in the RDC/MDC pathways, the combined risk for cancer of any type is high, with conversion rates higher than most 2WW pathways. Across the five MDC pilots in England, 241 cancers were diagnosed following 2961 referrals, with a conversion rate of 8.1% spread across multiple cancer sites. Of those cancers with stage data available, 25 (13.0%) were Stage I, 24 (12.4%) Stage II, 39 (20.2%) Stage III, and 105 (54.4%) Stage IV. RDCs, however, are resource intensive: for example, all patients referred to some RDCs undergo full body low-dose Computed Tomography (CT), blood tests, specialist review, with onward radiological and endoscopic investigations as indicated. A multi-cancer early detection test (MCED) that predicts the tissue of origin could both help establish cancer as a likely source of symptoms and direct further evaluation to the predicted tissue of origin.

1.3.2 GRAIL'S MCED TEST

GRAIL's Galleri™ MCED blood test is a qualitative, next-generation sequencing (NGS)-based screening test using cell-free DNA isolated from adult human peripheral whole blood. When a cancer signal is detected, the test also predicts the cancer signal origin. The test report describes one or two Cancer Signal Origins (CSOs). If the first CSO score is high (≥9.0), then only one CSOs is reported, otherwise the two top CSOs are reported. MCED has 21 possible CSOs: anus; bladder; urothelial tract; bone and soft tissue; breast; cervix; colon, rectum; head and neck; kidney; liver/bile duct; lung; lymphoid lineage; melanocytic lineage; myeloid lineage; neuroendocrine; ovary; pancreas, gallbladder; plasma cell lineage; prostate; stomach, oesophagus; thyroid gland; uterus. It is designed as a screening test and not to confirm a cancer diagnosis. Although the test result of "cancer signal detected" with Cancer Signal Origin may indicate the presence of cancer, further investigations to diagnose cancer are necessary in accordance with professional guidelines"

Preliminary evaluations of the GRAIL approach to blood markers were presented in 2018 and 2019. The Circulating Cancer Genome Atlas (CCGA) study (Clinicaltrials.gov identifier: NCT02889978) enrolled ~10,000 participants with cancer and 5,000 participants without a clinical diagnosis of cancer from medical institutions and networks throughout the United States and Canada. The pre-specified locked assay and classifier that was developed, analytically validated, and clinically validated in the CCGA study will be used in this study.

The investigational test system includes bioinformatic analysis pipeline software that uses the targeted methylation assay output to detect cancer and predict CSO, blood collection tubes (cell-free DNA blood collection tubes manufactured by Streck, Inc.), GRAIL's study test kit, and an NGS assay.

The first CCGA classification analysis (CCGA1) consisted of 2,800 participants, including 1,650 participants with newly diagnosed cancer who had not yet received treatment and 1,150 non-cancer participants. The prototype assays detected a highly specific, strong biological signal in cancer types that are typically not screened for and have low survival rates (five-year cancer-specific mortality rate of greater than 50 percent): lung, ovarian, pancreatic, liver, and oesophageal cancers. For these cancers in the CCGA1 Training set at 98% specificity, the sensitivity for stages I-III cancers (N=117) was 54% with the highest performing prototype assay, whole genome bisulfite sequencing. Sensitivity increased to 90% for stage IV cancers (N=81). Longitudinal follow-up has confirmed that three of eight participants who had an elevated signal but no cancer diagnosed at the time were later diagnosed with cancer suggesting that the signal indicated the presence of cancer before it was detectable by diagnostics used in clinical practice.

The second CCGA cohort (CCGA2) included ~4,800 participants and used a targeted methylation (Tme) cfDNA assay. This cohort served as the basis for the development of the GRAIL test classifier that distinguishes cancer from non-cancer and identifies cancer signal origin. CCGA2 included individuals presenting to clinical attention prior to cancer diagnosis with signs and/or symptoms ultimately resulting in a diagnosis of cancer. Non-cancer participants were defined by having no cancer at enrolment and non-cancer status confirmed at year one follow-up. This study demonstrated the ability of the test to achieve high specificity (99.5%, 95%CI 98.2-99.9%) in the non-cancer group, moderate sensitivity in those presenting to clinical attention (66.4%, 95%CI 62.2-70.3%), and high overall accuracy of cancer signal origin prediction (91.7%, 95% CI: 88.3-94.3%) in those presenting to clinical attention.

The third CCGA substudy was conducted in approximately 5,300 participants to validate updated versions of the classifiers for cancer signal detection and cancer signal origin prediction. The mean (SD) age in the cancer and non-cancer groups was 62.6 (11.76) and 56.2 (12.63) years, respectively. Specificity for cancer signal detection was 99.5% (1248/1254; 95% confidence interval: 99.0-99.8%). Overall sensitivity for cancer signal detection was 51.5% (1453/2823; 49.6-53.3%); sensitivity increased with stage (Stage I: 16.8% [14.5-19.5%], Stage II: 40.4% [36.8-44.1%], Stage III: 77.0% [73.4-80.3%], Stage IV: 90.1% [87.5-92.2%]). Stage I-III sensitivity was 67.6% (593/877; 64.4-70.6%) in a pre-specified set of 12 high-signal cancers (anus, bladder, colon/rectum, esophagus, head and neck, liver/bile duct, lung, lymphoma, ovary, pancreas, plasma cell neoplasm, and stomach) accounting for ~63% of annual US cancer deaths and was 40.7% (863/2118; 38.7-42.9%) in all cancers. Cancer signals were detected across >50 cancer types. Overall accuracy of signal origin prediction in true positives was 88.7% (87.0-90.2%).

Within this context, GRAIL's MCED blood test could complement the existing 2WW and RDC pathways by offering a novel approach to 2WW and non-2WW pathway selection for people presenting to their GP with symptoms that could be from cancer. Here, we evaluate the performance of the test in predicting cancer versus non-cancer as well as cancer signal origin for multiple cancers in secondary care cancer clinics. This includes cancers with high 5-year mortality that do not currently have a screening programme and some for which workup based

on symptoms is not yet standardised. Impact on management, time to diagnosis and resource utilisation are assessed within the NHS context.

GRAIL's Galleri™ MCED blood test is currently CE and UKCA marked as a screening test in an asymptomatic population and is registered with the MHRA as an IVD. The test indication does not include an indication in the symptomatic population This study is classified as a Performance Evaluation of an In-Vitro Diagnostic Device (PEIVDD) for the symptomatic population indication

1.4 Objectives

The SYMPLIFY study aims to evaluate the performance of GRAIL's MCED blood test in relation to a diagnosis of invasive cancer obtained within 12 months from initial referral.

Primary Objective

- To evaluate the performance of a MCED test for the detection of invasive cancer

Secondary Objectives

- To evaluate the performance of a MCED test by referral pathway (i.e. lung, upper GI, lower GI, gynae, and RDC) and cancer type and stage
- To evaluate the performance of a MCED test for the identification of cancer signal origin
- To evaluate the yield with MCED by referral pathway

Exploratory Objectives

- To evaluate the completeness of patient data collected from central databases
- To investigate if clinical parameters further optimise the performance of MCED test
- To evaluate the time to diagnostic resolution by referral pathway
- To estimate resource utilisation by referral pathway
- To evaluate the yield of non-cancer diagnoses following referral.
- To evaluate MCED test performance against GP suspected cancer site, based on 2WW clinic type selected at baseline.

2 Study Methods

2.1 Study design

This is a multi-centre, observational study with prospective collection and retrospective analysis of blood samples to evaluate the performance of a multi-cancer early detection test within the NHS in England and Wales.

The study will enrol 6000 participants over a period of approximately 3 months. Individuals who have been referred to a 2WW or RDC urgent cancer pathway by their GP will be invited to take part. Recruitment will take place in secondary care following referral, when patients attend for investigation. The anticipated breakdown by pathway is as follows:

- **lung 2WW** (n=500, expected cancer conversion rate = 16%); the majority of cancers diagnosed are expected to be lung cancer
- **gynae 2WW** (n=1000, expected cancer conversion rate = 4%); the majority of cancers diagnosed are expected to be ovarian cancer

- **upper GI 2WW** (n=2000, expected cancer conversion rate = 4%); the majority of cancers diagnosed are expected to be oesophagus, stomach, pancreas or liver cancers,
- **lower GI clinic** (n=2000, expected cancer conversion rate = 4%); the majority of cancers diagnosed are expected to be colorectal cancer
- **Rapid Diagnostic Centres** (RDC) (n=500, expected cancer conversion rate = 7%); the majority of cancers diagnosed are expected to be pancreatic, other upper GI, lung, colorectal, lymphoma, multiple myeloma, and renal tract.

Following informed consent, participants will be registered and assigned a study participant identification number. Approximately 40 mL of whole peripheral blood will be collected from all participants. The blood samples collected from participants will be shipped to a laboratory in the UK for processing to plasma and storage. As the MCED test will not be run until after the patient completes their investigative pathway, as the test requires clinical validation for a symptomatic use case, no individual results will be returned to the study participants or the clinicians responsible for their care.

Clinical information and demographic data will be collected from all participants via case report forms and NHS datasets. There are no protocol-required diagnostic procedures. Decisions regarding which specific diagnostic procedures should be performed and all medical decision making will be according to established clinical practice in the relevant clinical pathway.

The result of any diagnostic procedure(s) will be recorded until the date of diagnostic resolution, with patient level data collected from both participating hospital records and NHS datasets. Data will be collected within 3 months of enrolment to identify cancer diagnoses and serious disease outcomes, and at a later single time point between 6 and 9 months of enrolment for those without diagnostic resolution at 3 months. At 12 months, patient level data will be collected from primary and secondary care electronic health records and linked NHS datasets to capture cancers diagnosed later and healthcare utilisation following recruitment. Participants will therefore be involved only at their initial visit.

2.2 Index Test

The results from the MCED test for the detection of invasive cancer and the identification of cancer signal origin (CSO).

2.3 Reference – Diagnostic Resolution

Diagnostic resolution outcomes will be confirmed by clinical review of cases and will include:

- **Cancer diagnosis**: pathologic confirmation or other accepted criteria for confirmation of an invasive solid tumour or haematological malignancy excluding squamous cell carcinoma of the skin and basal cell carcinoma of the skin that are not metastatic, or other accepted criteria in the absence of pathology.
- **Non-cancer diagnosis**: diagnosis other than cancer at the conclusion of diagnostic evaluation for the presenting complaint, including carcinoma in situ and premalignant malignancies.
- **No diagnosis**: no diagnosis of cancer and no non-cancer condition diagnosed to explain the presenting complaint.

The clinical team at site will review the patient's entire clinical record for the recruited patient at the 3 and 9 month timepoints. If diagnostic resolution has been reached at the 3

month 'interim' timepoint there will be no need to review this participant at 9 months. If diagnostic resolution has not been reached at 3 months then review at 9 months will utilise any additional clinical information in the participants record to reach a decision about diagnostic resolution +/- diagnosis.

We expect most diagnoses to be made in the first two months and fewer to necessitate further follow-up (between 3 and 9 months) to reach diagnostic resolution. The primary analysis will occur when all data are collected and the database is locked. An interim analysis will occur based on the 3 month data post-enrolment of all participants.

2.4 Order of testing and independence of results

Blood samples that will form the basis of the MCED tests will be collected following informed consent. These results will not be available until after the patient completes their investigative pathway. Diagnostic resolution will be based on evaluation of multiple tests/pathways and, in relevant cases, confirmed by clinical review. No information regarding diagnostic resolution will be provided to the group carrying out the MCED analysis and therefore results from index and reference can be assumed to be independent.

2.5 Outcomes measures

2.5.1 Primary outcome

To evaluate the performance of a MCED test for the detection of invasive cancer, positive predictive value (PPV), negative predictive value (NPV), sensitivity, and specificity will be estimated for the complete analysis at 12 months of complete enrolment using diagnostic resolution as reference standard. An interim analysis of this outcome will also be carried out within 3 months of complete enrolment.

2.5.2 Secondary outcomes

To evaluate the performance of a MCED test for the detection of invasive cancer by referral pathway (i.e. lung, upper GI, lower GI, gynae, and RDC) and cancer type and stage, PPV, NPV, sensitivity, specificity and cancer signal origin accuracy will be estimated for the complete analysis at 12 months of complete enrolment using diagnostic resolution as reference standard. Cancer stage will be based on the TNM (Tumour Node Metastases) classification system where available, and a site-specific staging system otherwise. An interim analysis of this outcome will also be carried out within 3 months of complete enrolment.

To evaluate the performance of a MCED test for the identification of cancer signal origin label (see section 5 for definitions), positive predictive value (PPV), negative predictive value (NPV), sensitivity, specificity and cancer signal origin accuracy overall, by cancer site and by pathway selected by the GP will be estimated for the complete analysis at 12 months of complete enrolment using diagnostic resolution as reference standard. An interim analysis of this outcome will also be carried out within 6 months of complete enrolment.

To evaluate the yield with MCED by referral pathway the number of true positives/number of patients referred within each referral pathway will be obtained for the complete analysis at 12 months of enrolment using diagnostic resolution as reference standard. An interim analysis of this outcome will also be carried out within 3 months of complete enrolment.

2.5.3 Exploratory outcomes

To evaluate the completeness of patient data collected from central databases, the proportion of completed data fields, according to locally and centrally sourced inputs will be estimated locally within 3 months and by 9 months of enrolment, and centrally monthly from 3 through 12 months post enrolment.

To investigate if clinical parameters further optimise the performance of the MCED test, PPV, NPV, sensitivity and specificity based on outputs from a prediction model (see section 6) will be estimated at 12 months post enrolment using diagnostic resolution as reference standard.

To evaluate the time to diagnostic resolution by referral pathway, the days between enrolment and diagnostic resolution will be obtained at 12 months post enrolment.

To estimate resource utilisation by referral pathway, the number of encounters, tests, and referrals required to achieve diagnostic resolution will be obtained at 12 months post enrolment.

To evaluate the yield of non-cancer diagnoses following referral, the number of patients diagnosed with non-cancer/number of patients referred will be obtained at 12 months post enrolment

2.6 Target population

2.6.1 Study Participants

Participants referred to an RDC or relevant 2WW pathway to rule a cancer diagnosis in or out will be invited to participate in the study.

2.6.2 Inclusion Criteria

- Willing and able to give informed consent for participation in the study.
- Male or Female, aged 18 years or above.
- Referred to a RDC or a gynae, lung, upper GI or lower GI cancer 2WW pathway.

2.6.3 Exclusion Criteria

A potential participant may not enter the study if ANY of the following apply:

- has a history of invasive or haematological malignancy diagnosed within the previous 3 years
- has undergone definitive treatment for invasive or haematological malignancy in the last 3 years (adjuvant hormone therapy is permissible in this context).
- is taking cytotoxic or demethylating agents such as methotrexate
- previous or current participation in another GRAIL study. “Participation” is defined as having signed consent and provided a blood sample.

2.7 Sample size

The latest NHS England two week wait clinic data (from 2018) were used to estimate the distribution by cancer type and stage within each pathway. Sensitivity estimates by cancer type and stage for GRAIL v2.9 training and holdout, cross-validated data using isotonic regression were used at a specificity of 99.4% to estimate the expected PPV and NPV for a given sample size. It is expected that with a sample size of 6000 the PPV will be 86.8% (95% CI: 82.1%, 90.7%) and NPV will be 98.6% (95% CI: 98.3%, 98.9%). The anticipated contribution of each clinical pathway to the 6000 participants is described in section 2.

3 ANALYSIS – GENERAL CONSIDERATIONS

All confidence intervals presented will be 95% and two-sided. All applicable statistical tests will be two-sided. STARD (Bossuyt et al, 2015) reporting standards will be followed including a STARD flow diagram to summarise the flow of participants throughout the study including the number of withdrawals with reasons and the number of patients excluded with reasons.

3.1 DESCRIPTIVE STATISTICS

Participant characteristics will be summarised using descriptive statistics with figures and/or tables as appropriate. These summaries will be stratified by the MCED test result, referral pathway, and cancer type (see Appendix 2) and stage. For cancer types that have 10 or fewer observations, they will be grouped and summarized as "other."

Categories of data collection will include the following:

- Participant related identifiers (e.g., NHS number, DOB)
- Demographics (e.g., ethnicity, sex)
- Baseline clinical information (e.g., smoking, alcohol use, personal and family history of cancer, questions on cancer screening adherence)
- Referral information: Type of referral clinic and pathway, referral criteria
- Cancer and other diagnosis information at follow up timepoints: diagnostic resolution, tests completed as part of diagnostic work up, cancer diagnosis and associated tumour characteristics, and staging.

3.2 DEFINITION OF POPULATION FOR ANALYSIS

For the purposes of analysis, the following populations are defined:

- **Enrolled**: all consented participants
- **Clinically Evaluable**: consented participants who are clinically evaluable. Evaluability is defined as completing the standard investigative pathway to rule cancer in or out, with sufficient clinical data to assure the determination
- **Analysable**: all clinically evaluable participants with evaluable MCED test results
- **Diagnostic Resolution**: clinically evaluable patients with an analysable MCED test result whose diagnosis was resolved at the time of analysis.

3.3 POOLING OF SITES AND REFERRAL PATHWAYS

Primary analysis will be based on all data across recruitment sites and pathways. Secondary analysis will provide stratified results by referral pathways but across sites. No adjustment to deal with potential clustering due to recruitment sites is planned.

3.4 DATA MONITORING COMMITTEE AND INTERIM ANALYSES

No study specific monitoring committee will be engaged.

The Trial Management Group (TMG) will be responsible for day to day conduct of the study. The TMG will consist of the Chief (Chair) and Lead Investigators, Trials Unit representatives, a Sponsor representative and representatives from GRAIL.

The study will be overseen by the relevant oversight committees of the two trials units involved, taking into account input from the GRAIL Clinical Advisory Group

There will be one interim analysis at 3 months for the primary objective (only) and one at 6 months for the secondary objectives. These analyses are for information only and will not impact on the overall completion of the study. The rationale is to provide an early indication of the performance of MCED as required by the funder. A description of the outcomes that will be included in this interim analysis are found in section 2.4. Given that this is a Performance Evaluation of an In-Vitro Diagnostic Device (PEIVDD) no blinding of the study analyst is planned.

4 PRIMARY ANALYSIS

4.1 PRIMARY OUTCOME

The primary analysis is to evaluate the performance (sensitivity, specificity, PPV and NPV) of the MCED test for the detection of new invasive cancer based on target cancer conditions and cancer signal detected results of the MCED test.

We define target cancer conditions as:

- Invasive solid tumors, excluding non-metastatic basal cell carcinoma of the skin and squamous cell carcinoma of the skin
- Hematologic malignancies (with behavior code 3 based on ICD-O-3)

To get confirmation of target cancer condition, a tissue diagnosis documented in the EMR is required. This includes pathologic analysis of blood. Cytology is acceptable if it is supported by imaging. Imaging and AFP together are acceptable confirmation of hepatocellular carcinoma. Participants that do not have tissue diagnosis documented but with biochemical evidence or imaging evidence supporting an invasive cancer or hematologic malignancy will be labelled as 'probable' cancer condition cases. All confirmed and probable cancer condition cases will be included in the primary analysis. A sensitivity analysis will be carried out including only confirmed cases.

Two separate sets of estimates of test performance for the MCED test will be obtained: an early interim estimate which will be based on data captured within 3 months after enrolment and a late/complete estimate based on data at 12 months after enrolment to account for delayed diagnoses (including the 9-month and 12-month follow-up data).

Sensitivity: The proportion of participants with "signal detected" test results out of all participants with cancer diagnosis
Specificity: The proportion of participants with "signal not detected" results out of all participants with no cancer diagnosis (non-cancer and no diagnosis) at the time of the analysis
PPV: The proportion of participants with cancer diagnosed by diagnostic resolution out of all participants with "signal detected" test results
NPV: The proportion of participants with no cancer diagnosis (non-cancer diagnosis and no diagnosis) out of all participants with "signal not detected" results at the time of the analysis
Following FDA guidance (FDA 2007), all results, including failures and equivocal results, will be reported using tables, while analysis of test performance will be based on valid results only (analysable population). The MCED test performance parameters PPV, NPV, sensitivity, specificity will be evaluated overall and reported using point estimates along with 95% CIs. The interim analysis at 3 months will summarise the status of the full cohort but the calculation

of test performance will only include participants who have reached diagnostic resolution (e.g. cancer diagnosis vs. no cancer diagnosis (non-cancer and no diagnosis)) related to the initial cancer pathway referral. Two-sided 95% confidence intervals (CIs) will be constructed for proportion estimates using the Wilson (score) method (Wilson, 1927), unless otherwise specified. Tables and/or figures will be used to report these estimates with associated 95%CIs.

4.2 Handling missing data

Results of all tests (MCED and diagnostic resolution) will be recorded. Patterns of missingness for MCED will be investigated by tabulating patient characteristics and diagnostic resolution results. The primary analysis of MCED test performance will be based on valid results only (analysable population) and therefore no imputation will be carried out. If relevant patterns of missingness are observed, these will be included when discussing limitations of the primary analysis.

4.3 Handling multi-centre/clustered data

No pre-specified differences are expected between recruitment sites and therefore no statistical adjustment is planned for the primary analysis.

4.4 Multiple comparisons and multiplicity

No multiple testing/comparisons are expected and hence no adjustment is planned for the primary analysis.

4.5 Model assumptions

No modelling is planned and therefore no formal statistical assumptions are required for the primary analysis.

5 SECONDARY ANALYSIS

To meet the first secondary objective, we will stratify the performance (sensitivity, specificity, PPV and NPV) of the MCED test for the detection of invasive cancer by referral pathway (i.e. lung, upper GI, etc.) and sensitivity for cancer signal detected by cancer type (Appendix 2) and clinical stage. Two separate sets of estimates of test performance for the MCED test will be obtained: an early interim estimate (at 3 months) which will be based on data captured within 3 months after enrolment and a late/complete estimate based on data at 12 months after enrolment to account for delayed diagnoses (including the 9-month and 12-month follow-up data).

To meet our second secondary objective, a comparison will be made between the top-one predicted signal origin label from the MCED report (one of 22 possible labels (including no-cancer); see Appendix 1) and two different/distinct reference standards: (i) clinical outcome in NHS services and (ii) CSO mapping by clinical scientists at GRAIL. We will repeat the overall analysis stratified by the participant's general practitioner's (GP) choice of referral pathway, resulting in five separate subgroup analyses (Lung, Upper GI, Lower GI, Gynae, RDC).

Accuracy of signal origin prediction is defined as the proportion of participants with a correct predicted signal origin label among participants with the relevant reference standard. Specifically, the following accuracy of signal origin prediction calculations are used in this study:

- Main overall accuracy of signal origin prediction is defined as the proportion of participants with a correct top-one predicted signal origin label among those with the relevant reference standard.
- As a sensitivity analysis, secondary overall accuracy of signal origin prediction is defined as the proportion of participants with a correct top-one or top-two predicted signal origin label among those with the relevant reference standard (see section 7 for further details).
- As a second sensitivity analysis, we will carry out this accuracy of signal origin prediction evaluation focusing only on cancers detected based on the relevant reference standard AND those with a cancer signal detected in the results of the MCED test.

For **(i) clinical outcomes in NHS services**, we will follow a modified National Disease Registration Service (NDRS) Routes to Diagnosis classification based on ICD-10 and ICD-O3 codes to map all cancer diagnoses into one of 25 cancer categories (plus no-cancer diagnosis; see Appendix 3). The results for this analysis will be presented in a table comparing the clinical outcomes in NHS services label (X-axis) to the top one predicted signal origin label (Y-axis) constructed similar to that in Table 5.1 (by clinical outcome category). A mock version of this table with labels is presented in Appendix 5.

Table 5.1. Summary for Signal Origin Prediction vs. clinical outcomes in NHS services

		Clinical outcomes in NHS services				Total
		CO_1	CO_2	…	CO_26	
Top-one Predicted Signal Origin	PSO_1	N_1,1	N_1,2	…	N_1,26	PS_1
	PSO_2	N_2,1	N_2,2			PS_2
	...	…		…		...
	PSO_22	N_22,1			N_22,26	PS_22
Total		C_1	C_2	...	C_26	

Denote the clinical outcome in NHS services label for category i as CO_j (j = 1, 2, …, 25); CO_22 = no-cancer detected, and the corresponding predicted signal origin label as PSO_i (i = 1, 2, …, 22); PSO_22= no-cancer. Denote the total number of participants with a clinical outcome CO_j as C_j (j = 1, 2, …, 26). Denote the total number of participants with a predicted signal origin label PSO_i as PS_i (i = 1, 2, …, 22). Denote the total number of participants with a top-one predicted signal origin label PSO_i and clinical outcome in NHS services CO_j as N_i,j (i = 1, 2, …, 22; j = 1, 2, …, 26). Denote N_i,i as a ‘correct’ top-one predicted signal origin label (e.g. CO’s 23, 24, 25, and 26 not mapping to any PSO).

The following measures are calculated from the table and presented as point estimates with associated 95%CIs:

- **Concordance of CSO with clinical outcome in NHS services** is defined as the proportion of participants with a 'correct' top-one predicted signal origin label among true positive participants with a particular clinical outcome in NHS services. It is estimated as the proportion of correct diagnoses in each column of Table 5.1, i.e., 100% * N_i,i /C_i (i = 1, 2, …, 22); excluding CO's 23, 24, 25, and 26 as these are not mapped to any PSO).
- **Concordance of clinical outcome in NHS services with CSO i**s defined as the proportion of participants whose predicted signal origin label matches their target clinical outcome in the NHS services among true positive participants with a particular predicted signal origin label. It is estimated as the proportion of correct diagnoses in each row of Table 5.1, i.e., 100% * N_i,i /PS_i (i = 1, 2, …, 22).

For (ii) CSO mapping by clinical scientists at GRAIL, multi-class confusion matrix comparing the target signal origin label (X-axis) to the top one predicted signal origin label (Y-axis) is constructed (Table 5.2) among true positive participants with a single trained target signal origin label. The target signal label "prediction only" is excluded from this confusion matrix analysis and summarised separately. Similarly, results where no 'predicted signal origin' were provided will be presented separately. A mock version of this table with labels is presented in Appendix 6.

Table 5.2. Confusion Matrix for Signal Origin Prediction vs. CSO mapping by clinical scientists at GRAIL

		Target Signal Origin				Total
		SO_1	SO_2	…	SO22	
Top-one Predicted Signal Origin	PSO_1	M_1,1	M_1,2	…	M_1,22	PS_1
	PSO_2	M_2,1	M_2,2			PS_2
	...	…		…		...
	PSO_22	M_21,1	M_21,2		M_22,22	PS_22
Total		S_1	S_2	...	S_22	

Denote the signal origin label that corresponds to a trained signal origin label as SO_i (i = 1, 2, …, 22); SO_22= no-cancer, and the corresponding predicted signal origin label as PSO_i (i = 1, 2, …, 22); PSO_22= no-cancer. Denote the total number of participants with a target signal origin label SO_i as S_i (i = 1, 2, …, 22). Denote the total number of participants with a predicted signal origin label PSO_i as PS_i (i = 1, 2, …, 22). Denote the total number of participants with a top-one predicted signal origin label PSO_i and signal origin label SO_j as M_i,j (i = 1, 2, …, 22; j = 1, 2, …, 22). Denote M_i,i as a 'correct' top-one predicted signal origin label.

The following measures are calculated from the confusion matrix and presented as point estimates with associated 95%CIs:

- **Accuracy of signal origin prediction by signal origin** is defined as the proportion of participants with a 'correct' top-one correct diagnoses in each column of the confusion matrix, i.e., 100% * $M_{i,i}$ /S_i (i = 1, 2, …, 22). See Table 5.2.
- P**recision of signal origin prediction by signal origin i**s defined as the proportion of participants whose predicted signal origin label matches their target signal origin label among true positive participants with a particular predicted signal origin label. It is estimated as the proportion correct diagnoses in each row of the confusion matrix, i.e., 100% * $M_{i,i}$ /PS_i (i = 1, 2, …, 22). See Table 5.2.

Performance by clinical pathway:
As stated above, to analyse the performance of the signal origin prediction by clinical pathway, we will repeat the analysis based on the two different/distinct reference standards: (i) clinical outcome in NHS services and (ii) CSO mapping by clinical scientists at GRAIL (see above) stratified by the participant's general practitioner's (GP) choice of referral pathway, resulting in five separate subgroup analyses (Lung, Upper GI, Lower GI, Gynae, RDC). We will report the same measures as in the non-stratified analyses: a) Concordance of CSO with clinical outcome in NHS, b) Concordance of clinical outcome in NHS services with CSO, c) Accuracy of signal origin prediction by signal origin, and d) Precision of signal origin prediction by signal origin.

A 6 month CSO interim analysis will use the same locked dataset as the 3 month interim analysis. This is to allow CSO mapping to be conducted by GRAIL using the 3 month data after the 3 month dataset is locked by the University and the test results have been received from GRAIL and locked by the University.
To meet our third secondary objective, yield will be estimated based on the number of cancer cases that were also MCED test positive (true positives) out of the total tested (i.e. analysable population). Yield estimates will be presented overall, and by referral pathway using point estimates and 95%CI. This analysis will be included in the 3-month interim analysis as well as the final analysis.

6 EXPLORATORY ANALYSIS

A key output for the study will be an analysis of the completeness and quality of cancer diagnostic pathway data gathered from central NHS databases. Datapoints collected locally within 3 months and by 9 months of enrolment will be compared to data collected centrally monthly from 3 through 12 months post enrolment. The completeness, by capture method, and concordance between methods of each data field will be summarised descriptively. This will inform the potential to use central data capture for future large-scale studies in the field, as well as identify areas for improvement in central data linkage.

We will also investigate if patient demographic characteristics, referral information, and other relevant clinical parameters allow for further optimisation of the performance of the MCED test based on stratification and multivariable models. Assuming that cancer detection rate of 5%, we can expect (6000*5%) 300 cancers or events which would allow us to explore between 15-30 variables including the MCED test using 10 -20 events per variable heuristic. Logistic regression will be used with model specification based on careful preselection of candidate predictors guided by subject knowledge combined with automated backward

selection to remove the least significant candidate predictors. Bootstrap validation will be used to assess magnitude for optimism in performance of the model. Bootstrap is preferred over split sample approaches as all of the data is used for both model development and testing, and appropriately reflect all sources of model uncertainty including model selection. These models will be used as hypothesis generating only and will require validation using new and independent data.

The time from enrolment to diagnosis will be analysed overall and by cancer site, cancer stage, and referral pathway, using the survival methods (e.g. Kaplan-Meier method) with censoring based on death, withdrawal or loss to follow-up.

Serious disease yield will also be estimated based on the number serious disease cases diagnosed out of the total enrolled.

To evaluate MCED test performance against GP suspected cancer site, we will use information collected at baseline on 2WW clinic type (e.g. lung, ovarian, etc) based on the referral criteria selected by the GP and compare against MCED test CSD and CSO.

Health Economic Analysis

Health Outcome Measures

Cancer outcomes data will be extracted from hospital records at 3 and 9 months post enrolment and linked to routine health service use data at 12 months from the clinical study. Our initial outcome measure would include simply cancer cases detected. However, our primary outcome measure for the cost-effectiveness analysis will be quality-adjusted life years (QALYs), estimated using the EuroQol EQ-5D-5L which will be taken from literature estimates in this observational study, with a view to asking patients to complete actual EQ5D questions in a future interventional study. The EuroQol EQ-5D-5L questionnaire is advocated for use in economic evaluations by NICE, to measure health related quality of life (HRQoL). The questionnaire is a generic measure of health outcome covering five dimensions: mobility, self-care, usual activities, pain/discomfort, and anxiety/depression.
Established guidelines on the conduct of economic evaluations set out by NICE are similar to processes for undertaking economic evaluations in other countries, which will help make the results of the UK economic evaluation generalise to other settings. This early health economic analysis will also contribute to supporting further health economic assessment in a second phase interventional study.

Resource Use and Costs

Our analysis will include determining the healthcare resources and costs required for the MCED pathway compared to the standard care pathways for these patient groups. To estimate the healthcare resources and associated costs for the MCED approach compared to current practice, resource use information will be extracted from the study CRFs and hospital episode statistics (HES) data and primary care databases. We will quantify the number and type of clinical encounters in terms of consultations, tests, and referrals in primary and secondary care. This would include the costs of alternative diagnostic tests, primary care and secondary care visits and contacts (GP, hospital A&E, out-patient and inpatient care).

7 SENSITIVITY ANALYSIS

For the primary and secondary analyses, we will carry out sensitivity analyses at 12 months to exclude those participants recruited that have been identified as having recurrent cancers to focus only on new cancers. As the majority of individuals with cancer recurrence in the NHS are managed through a different pathway (through secondary care) to the one used for recruitment in this study, we expect only a small proportion of the participants recruited to this study to be excluded in this analysis. For the primary analysis, we will carry out a sensitivity analysis based only on confirmed cases (see section 4.1).

For our second secondary objective, to evaluate the performance of a MCED test for the identification of cancer signal origin, we will carry out a sensitivity analysis as part of a secondary overall accuracy of signal origin prediction. This is defined as the proportion of participants with a correct top-one or top-two predicted signal origin label as opposed to only the top-one (see section 5). In this calculation, if any of the top-one and top-two predicted signal origin labels match the target signal origin label, then it is considered a correct prediction. For those cancer cases where neither of the top-two that are an adequate match, the top-one will be used for classification and display purposes. As a second sensitivity analysis, we will carry out this accuracy of signal origin prediction evaluation focusing only on cancers detected based on the relevant reference standard AND those with a cancer signal detected in the results of the MCED test.

A final sensitivity analysis will be carried out for our first secondary objective, to determine performance (sensitivity, specificity, PPV and NPV) of the MCED test for the detection of invasive cancer by referral pathway (i.e. lung, upper GI, etc.) and sensitivity for cancer signal detected by cancer type as determined by GRAIL (see Appendix 7), instead of the NCRAS-RTD classification.

8 SUBGROUP ANALYSES

No subgroup analyses beyond those specified above are currently planned.

9 ADDITIONAL EXPLORATORY ANALYSIS

No additional exploratory analyses are currently planned.

10 SAFETY ANALYSIS

As this is a Performance Evaluation of an In-Vitro Diagnostic Device (PEIVDD) no safety analysis is expected to be carried out.

10.1 ADVERSE EVENTS

As this is a Performance Evaluation of an In-Vitro Diagnostic Device (PEIVDD) no adverse events due to the study are expected.

11 VALIDATION

No further validation is currently planned.

12 CHANGES TO THE PROTOCOL OR PREVIOUS VERSIONS OF SAP

First version of SAP based on SYMPLIFY_Protocol_v1.0_20May2021.
Second version of SAP (17 Dec 2021) based on SYMPLIFY_Protocol_v1.1_08Nov2021_BN_GRAIL – changes include:

- Modification of primary outcome to focus only on performance of MCED result for identification of invasive cancer (based on changes to the protocol)
- Addition of secondary objective to include accuracy of MCED result with target signal origin label (based on changes to the protocol)
- Extended definition of Diagnostic resolution
- Target signal origin label identified by three methods: (i) clinical outcome in NHS services, (ii) CSO mapping by clinical scientists at GRAIL (iii) the participant's general practitioner's (GP) choice of referral criteria
- Use of bootstrapping instead of split-data for model optimisation in exploratory analysis
- Inclusion of two sensitivity analysis:
    - Redefining target population based on 'new cancers' only
    - Analysis of MCED result based on top-two predicted cancer (instead of just top-predicted)

Third version of SAP (13 April 2022) based on SYMPLIFY_Protocol_v2_19 Jan 2022– changes include:

- Inclusion of target cancer conditions and definition 'confirmed cancers' and 'probable cancers' based on information on tissue diagnosis (see section 4.1).
- Modification of (secondary) analysis of target signal origin label compared to (i) clinical outcome in NHS services and (iii) the participant's general practitioner's (GP) choice of referral criteria. These are now based on concordance of target signal origin and these two measures.
- Inclusion of one sensitivity analysis extra: primary analysis based on 'confirmed cancers' only.
- Appendices added to support description of secondary analyses.

Fourth version of SAP (6 May 2022) based on SYMPLIFY_Protocol_v2_19 Jan 2022– changes include:

- Modification of (secondary) analysis of target signal origin now compared to two reference criteria: (i) clinical outcome in NHS services and (ii) (ii) CSO mapping by clinical scientists at GRAIL. The third comparison ((iii) the participant's general practitioner's (GP) choice of referral criteria) modified to a stratified analysis by the participant's general practitioner's (GP) choice of referral criteria using the two reference criteria above, instead of the previously proposed concordance analysis.
- Clarification of the categories expected for (i) clinical outcome in NHS services added into Appendix 3.
- Added Appendix 5 which includes templates of tables for CSO reporting.

Fifth version of SAP (26 May 2022) based on SYMPLIFY_Protocol_v2_19 Jan 2022– changes include:

- Addition of a second type of sensitivity analysis for the second secondary analysis based only on those that have a positive cancer diagnosis by the appropriate reference standard AND a positive cancer signal detected in the results of the MCED test.
- Change of Appendix 2 to include the different cancer types.

Sixth version of SAP (13 June 2022) based on SYMPLIFY_Protocol_v2_19 Jan 2022– changes include:

- Change to text to reflect GRAIL's GalleriTM MCED blood test now currently CE and UKCA marked as a screening test in an asymptomatic population and is registered with the MHRA as an IVD.
- Added exploratory analysis of MCED test compared to GP suspected cancer site.
- Addition of a third type of sensitivity analysis for the first secondary analysis based on cancer type as determined by GRAIL (see Appendix 7), instead of the NCRAS-RTD classification.
- Addition of Appendix 7.

13 References

Bossuyt PM, Reitsma JB, Bruns DE, Gatsonis CA, Glasziou PP, Irwig L, Lijmer JG, Moher D, Rennie D, de Vet HC, Kressel HY, Rifai N, Golub RM, Altman DG, Hooft L, Korevaar DA, Cohen JF; STARD Group. STARD 2015: an updated list of essential items for reporting diagnostic accuracy studies. BMJ. 2015;351:h5527. doi: 10.1136/bmj.h5527.

CADEAS. Available at: http://www.ncin.org.uk/local_cancer_intelligence/cadeas#covid-19. (Accessed: 29th January 2021)

Food and Drug Administration (FDA). Statistical Guidance on Reporting Results from Studies Evaluating Diagnostic Tests - Guidance for Industry and FDA Staff. US Department of Health and Human Services. March 2007. Available at: https://www.fda.gov/regulatory-information/search-fda-guidance-documents/statistical- guidance-reporting-results-studies-evaluating-diagnostic-tests-guidance-industry-and-fda. [Accessed 6th May 2021].

Miller KD, et al. Cancer Treatment and Survivorship Statistics, 2016. CA Cancer J Clin. 66, 271-289 (2016).

Rapid Diagnostic Centres: Vision and 2019/20 Implementation Specification.

Thompson M et al. Have large increases in fast track referrals improved bowel cancer outcomes in UK? BMJ **371**, (2020).

Wilson, E. B. (1927). "Probable inference, the law of succession, and statistical inference". Journal of the American Statistical Association. 22 (158): 209 212. doi:10.1080/01621459.1927.10502953. JSTOR 2276774.

World Health Organization. (2017). Guide to cancer early diagnosis. World Health Organization. https://apps.who.int/iris/handle/10665/254500.

Zhou Y et al. Variation in ‘fast-track’ referrals for suspected cancer by patient characteristic and cancer diagnosis: Evidence from 670 000 patients with cancers of 35 different sites. Br J Cancer **118**, 24–31 (2018).

APPENDIX 1. List of Signal Origin Labels

Signal origin labels that were used in the signal origin classifier training are listed below:

Signal Origin Label as specified on the V2 Signal Origin Reporting Labels Specification	Signal Origin Name as displayed on the Test Report
anus	Anus
bladder_and_urothelial	Bladder, Urothelial Tract
breast	Breast
cervix	Cervix
colon_rectum	Colon, Rectum
head_and_neck	Head and Neck
kidney	Kidney
liver_bileduct	Liver, Bile Duct
lung	Lung
lung_net_hg_net*	Neuroendocrine Cells of Lung or other Organs
lymphoid_neoplasm	Lymphoid Lineage
melanoma	Melanocytic Lineage
myeloid_neoplasm	Myeloid Lineage
ovary	Ovary
pancreas_gallbladder	Pancreas, Gallbladder
plasma_cell_neoplasm	Plasma Cell Lineage
prostate	Prostate
sarcoma	Bone and Soft Tissue
thyroid	Thyroid Gland
upper_gi	Stomach, Esophagus
uterus	Uterus
No-cancer detected	Cancer signal not detected

*lung neuroendocrine or other high grade neuroendocrine

APPENDIX 2. ANTICIPATED CANCER TYPES BASED ON NCRAS-RTD CLASSIFICATION

N	Cancer Type
1	Anus
2	Bladder
3	Breast
4	Cancer of Unknown Primary
5	Cervix
6	Central Nervous System
7	Colon/Rectum
8	Oesophagus
9	Gallbladder
10	Head and Neck
11	Kidney
12	Liver/Bile-duct
13	Lung
14	Lymphoid Leukemia
15	Lymphoma
16	Melanoma of skin
17	Myeloid Neoplasm [1]
18	Non-melanoma skin cancer
19	Other
20	Ovarian
21	Pancreas
22	Plasma Cell Neoplasm
23	Prostate
24	Sarcoma (Bone and soft tissue)
25	Stomach
26	Thyroid
27	Urothelial Tract
28	Uterus

[1] AML or CML

APPENDIX 3. LIST OF CLINICAL OUTCOME IN NHS SERVICES BASED ON NCRAS-RTD CLASSIFICATION

**Clinical Outcome in NHS Services**
1. Head and Neck
2. Oesophagus, Stomach
3. Colon, Rectum
4. Anus
5. Liver, Bile Duct
6. Pancreas, Gallbladder
7. Lung
8. Melanocytic Lineage (Melanoma of skin)
9. Breast
10. Cervix
11. Uterus
12. Ovary
13. Prostate
14. Kidney
15. Bladder, Urothelial Tract
16. Thyroid Gland
17. Myeloid Lineage
18. Lymphoid Lineage
19. Plasma Cell Lineage
20. Bone and Soft Tissue
21. Neuroendocrine
**Additional Clinical Groups (not part of GRAIL CSO)**
22. No-Cancer diagnosed
23. Central Nervous System

24. Non-melanoma skin cancer
25. Cancer unknown primary
26. Other

APPENDIX 4: SIGNAL ORIGINS AND ASSOCIATED SUBCATEGORIES

SIGNAL ORIGIN	SUBCATEGORIES
Head and Neck	Oropharynx, Hypopharynx, Nasopharynx, Larynx, Lip and Oral Cavity (including Oral Tongue), Nasal Cavity, Paranasal Sinuses, Major Salivary Glands
Colon, Rectum	Colon, Rectum, Appendix
Anus	Anus
Liver, Bile Duct	Liver, Intrahepatic Bile Duct
Pancreas, Gallbladder	Pancreas, Extrahepatic Bile Duct, Gallbladder
Lung	Lung, Bronchus
Melanocytic Lineage	Melanocytic Lineage
Breast	Breast
Cervix	Cervix
Uterus	Uterus
Ovary	Ovary, Fallopian Tube, Primary Peritoneum
Prostate	Prostate
Kidney	Kidney
Bladder, Urothelial Tract	Bladder, Renal Pelvis, Ureter, Urethra
Thyroid Gland	Thyroid Gland
Myeloid Lineage	Myeloid Lineage
Lymphoid Lineage	Lymphoid Lineage
Plasma Cell Lineage	Plasma Cell Lineage
Bone and Soft Tissue	Skeletal Muscle and Other Connective Tissue, Vascular Tissue, Bone and Cartilage
Stomach, Esophagus	Stomach, Esophagus
Neuroendocrine Cells of Lung or Other Organs	Neuroendocrine Cells of Lung or Other Organs

Effective

APPENDIX 5: SHELL TABLE TO PRESENT MCED CSD AND CSO RESULTS VS NHS OUTCOME FOR ALL PARTICIPANTS RECRUITED. THIS TABLE COULD BE REPLICATED FOR EACH CLINICAL PATHWAY.

			NHS Outcome																										
			Cancer																									No Cancer	Total
MCED Result	CSD+	n	H&N	Oes / Sto	Colorectal	Anus	Liver / BD	Panc / GB	Lung	Melano	Breast	Cervix	Uterus	Ovary	Prostate	Kidney	Blad / Uro	Thyroid	Myeloid	Lymphoid	Plasma	Bone & ST	NET	CNS	Skin	CUP	Other	n	N
		H&N																											
		Oes / Sto																											
		Colorectal																											
		Anus																											
		Liver / BD																											
		Panc / GB																											
		Lung																											
		Melanoma																											
		Breast																											
		Cervix																											
		Uterus																											
		Ovary																											
		Prostate																											
		Kidney																											
		Blad / Uro																											
		Thyroid																											
		Myeloid																											
		Lymphoid																											
		Plasma																											
		Bone & ST																											
		NET																											
	CSD-	n																										n	N
Total			N																									N	N

APPENDIX 6: SHELL TABLE TO PRESENT MCED CSD AND CSO RESULTS VS CSO MAPPING BY CLINICAL SCIENTISTS AT GRAIL FOR ALL PARTICIPANTS RECRUITED. THIS TABLE COULD BE REPLICATED FOR EACH CLINICAL PATHWAY.

		GRAIL Mapping																						Total
		Cancer																					No Cancer	
MCED Result	CSD+	n	H&N	Oes / Sto	Colorectal	Anus	Liver / BD	Panc / GB	Lung	Melano	Breast	Cervix	Uterus	Ovary	Prostate	Kidney	Blad / Uro	Thyroid	Myeloid	Lymphoid	Plasma	Bone & ST	NET	n	
		H&N																							
		Oes / Sto																							
		Colorectal																							
		Anus																							
		Liver / BD																							
		Panc / GB																							
		Lung																							
		Melanoma																							N
		Breast																							
		Cervix																							
		Uterus																							
		Ovary																							
		Prostate																							
		Kidney																							
		Blad / Uro																							
		Thyroid																							
		Myeloid																							
		Lymphoid																							
		Plasma																							
		Bone & ST																							
		NET																							
	CSD-	n																						n	N
Total		N																						N	N

APPENDIX 7 ANTICIPATED CANCER TYPES BASED ON GRAIL CLASSIFICATION

N	Cancer Type
1	Anus
2	Bladder
3	Breast
4	Cervix
5	Colon/Rectum
6	Esophagus
7	Gallbladder
8	Head and Neck
9	Kidney
10	Liver/Bile-duct
11	Lung
12	Lymphoid Leukemia
13	Lymphoma
14	Melanoma
15	Myeloid Neoplasm [1]
16	Ovary
17	Pancreas
18	Plasma Cell Neoplasm
19	Prostate
20	Sarcoma
21	Stomach
22	Thyroid
23	Urothelial Tract
24	Uterus

[1] AML or CML
