# Supplementary material for: Diagnostic Implications of Multi-Cancer Early Detection Testing in the Investigation of Cancer Symptoms: An Exploratory Retrospective Analysis of the SYMPLIFY Study
Source: Lancet Reg Health Eur. 2026 May 28;66:101720. doi: 10.1016/j.lanepe.2026.101720 (PMC13235408; doi:10.1016/j.lanepe.2026.101720)
Supplement: Additional Follow Up Registry [file mmc4.pdf]

Primary Care
Clinical Trials Unit

STATISTICAL ANALYSIS PLAN

SYMPLIFY

Study title: SYMPLIFY – Observational study to assess a multi-cancer early detection test in individuals referred with signs and symptoms of cancer

APPENDIX for Statistical Analysis Plan

Exploratory objective:

To assess the diagnostic accuracy of the MCED test using diagnoses within 2 years

Version: 1.0, 8th September 2025

Author: Pradeep Virdee

VERSION HISTORY

Version:	Version Date:	Changes:
0.1	19th August 2024	N/A – this is the first version.
0.2	27th May 2025	Cancer now defined as cancer diagnosis within 2 years on either site or registry data. Added sensitivity analysis looking at cancer within 2 years from registry data only.
0.3	30th May 2025	Included all diagnostic accuracy measures, not just the PPV. Added further analyses, such as time-to-diagnosis
0.4	15th July 2025	Added details on eligible cancers. There is now two main outcome definitions, plus additional definitions for different follow-up windows. The analysis strategy now includes an analysis that accounts for multiple cancer diagnoses per patient.
0.5	8th September 2025	Updated to reflect that the analysis will be incorporated as additional results in the off-target/MCED benefit paper, instead of being a standalone analysis

1 Objective

To assess the diagnostic accuracy of the MCED test using diagnoses within 2 years.

2 Outcome

Only cancers with an ICD-10 code starting with ‘C’ or D45–D46.9, D47.3, or D47.4 and an ICD-O-3 behaviour code of ‘3’ will be included. Non-melanoma skin cancers (C44) and stage 0 cancers will be excluded. We may use two main outcome definitions:

1. Two-year cancer diagnosis (yes/no), defined as any cancer diagnosis over the two-year follow-up period recorded in the <u>registry</u> dataset.
2. Two-year cancer diagnosis (yes/no), defined as any cancer diagnosis over the two-year follow-up period recorded in either <u>site or registry</u> datasets.

We may additionally assess cancer diagnosis over time or at 9, 12, 15, 18, and 21 months of follow-up. Furthermore, we may assess only those cancers that are considered congruent with the presenting symptoms, referral pathway, and/or MCED results.

3 Analysis strategy

Participant data will be summarised using descriptive statistics, including counts with proportions for categorical data and mean with standard deviation and/or median with range/interquartile range for continuous data. This includes reporting congruency between symptom, pathway, MCED, and cancer diagnosis.

For each outcome separately, we may derive the sensitivity, specificity, positive predictive value (PPV), and negative predictive value (NPV) (with 95% confidence intervals (CIs)) of the MCED test for two-year cancer risk. We may crosscheck diagnostic accuracy classifications (true/false positive/negative) from the original primary analysis, which used 9-month site-collected data, with those based on the updated registry dataset and describe whether the additional registry cancers are congruent with presenting symptoms, referral pathway chosen, and the initial MCED result (CSD and CSO). We may summarise the time from registration to diagnosis, site, and stage of cancers.
